# Representative Sinusoids for Hepatic Four-Scale Pharmacokinetics Simulations

**DOI:** 10.1371/journal.pone.0133653

**Published:** 2015-07-29

**Authors:** Lars Ole Schwen, Arne Schenk, Clemens Kreutz, Jens Timmer, María Matilde Bartolomé Rodríguez, Lars Kuepfer, Tobias Preusser

**Affiliations:** 1 Fraunhofer MEVIS, Bremen, Germany; 2 Computational Systems Biology, Bayer Technology Services, Leverkusen, Germany; 3 Aachen Institute for Advanced Study in Computational Engineering Sciences, RWTH Aachen University, Aachen, Germany; 4 Freiburg Center for Data Analysis and Modeling (FDM), Institute of Physics, University of Freiburg, Freiburg, Germany; 5 BIOSS Centre for Biological Signalling Studies, University of Freiburg, Freiburg, Germany; 6 Clinic for Internal Medicine II/Molecular Biology, University of Freiburg Medical Center, Freiburg, Germany; 7 Institute of Applied Microbiology, RWTH Aachen University, Aachen, Germany; 8 Jacobs University, Bremen, Germany; University College London, UNITED KINGDOM

## Abstract

The mammalian liver plays a key role for metabolism and detoxification of xenobiotics in the body. The corresponding biochemical processes are typically subject to spatial variations at different length scales. Zonal enzyme expression along sinusoids leads to zonated metabolization already in the healthy state. Pathological states of the liver may involve liver cells affected in a zonated manner or heterogeneously across the whole organ. This spatial heterogeneity, however, cannot be described by most computational models which usually consider the liver as a homogeneous, well-stirred organ.

The goal of this article is to present a methodology to extend whole-body pharmacokinetics models by a detailed liver model, combining different modeling approaches from the literature. This approach results in an integrated four-scale model, from single cells via sinusoids and the organ to the whole organism, capable of mechanistically representing metabolization inhomogeneity in livers at different spatial scales. Moreover, the model shows circulatory mixing effects due to a delayed recirculation through the surrounding organism.

To show that this approach is generally applicable for different physiological processes, we show three applications as proofs of concept, covering a range of species, compounds, and diseased states: clearance of midazolam in steatotic human livers, clearance of caffeine in mouse livers regenerating from necrosis, and a parameter study on the impact of different cell entities on insulin uptake in mouse livers.

The examples illustrate how variations only discernible at the local scale influence substance distribution in the plasma at the whole-body level. In particular, our results show that simultaneously considering variations at all relevant spatial scales may be necessary to understand their impact on observations at the organism scale.

## Introduction

The liver plays a pivotal role for metabolization and detoxification in the mammalian body. The connection between the whole organism and the liver is mainly given by blood flow: blood is supplied to the liver via two vascular systems, portal vein (PV) and hepatic artery (HA), blood is drained via the hepatic vein (HV). The PV and HA provide approximately 75% and 25% of the inflow, respectively [1]. Besides the blood flow, additional exchange between organ and organism is given by bile [1] and lymph [2] flow.

Clearance contributions of different organs are typically analyzed by pharmacokinetics (PK) models [3–5]. In particular, specific models for liver clearance were developed [3, 6].

One major challenge for PK simulations, in particular for relevant diseased states, is the presence of spatial inhomogeneity and multiple scales in time and space that need to be accounted for appropriately. The most prominent spatial scales in the liver are:

1. individual hepatocytes performing most of the metabolization of diameter 20 to 40μm [1] in humans, 23.3 ± 3.1 μm in mice [7];
2. sinusoids and space of Disse along which the blood gets in contact with the hepatocytes;
3. lobuli consisting of multiple sinusoids draining to the same central vein. Lobuli have a diameter of 1 to 1.3 mm and a depth of 1.5 to 2 mm in humans [1]. Their radius is 284.3 ± 56.9 μm in mice [7]. The radius of a lobulus is thus about 19 hepatocyte diameters in humans and 12 in mice;
4. the liver and its lobes, organized in many lobuli, supplied and drained by the main major branchings of the hepatic vascular structures. The total liver volume is about 1.5 l in humans [1] and approximately 1.1 ml in mice [8]. Human livers are classically divided in two lobes or eight segments [9], but there is no consensus in the literature on the subdivision and its terminology, neither for mice [10] nor for humans [11].

**Fig 1:**
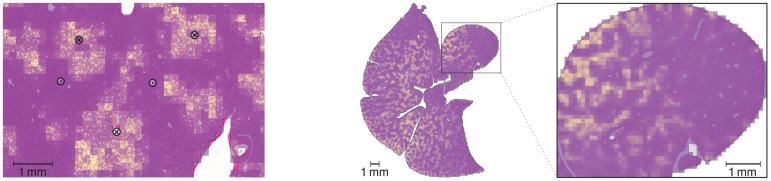
Steatosis Inhomogeneity. The *left* image shows a histological image of a human liver with selected portal fields and central veins marked as ⊙ and ⊗, respectively. The *right* image shows a histological whole-slide scan of a steatotic mouse liver and a zoom to one lobe. Macrovesicular steatosis, i.e., lipid accumulations of diameter larger than hepatocyte nuclei, was quantified in all cases using an image analysis method based on [14] and visualized as an overlay to the histological images, using a color map from violet to yellow indicating low to high steatosis. The left example shows a pericentrally zonated state of steatosis. The right example shows both organ-scale and lobe-scale heterogeneity in the steatosis distribution in addition to a periportal zonation not clearly visible at this magnification. The human image data is by Serene Lee and Wolfgang Thasler, Department of General, Visceral, Transplantation, Vascular and Thoracic Surgery Ludwig Maximilians University Munich Medical Center; the mouse image data is by Uta Dahmen, Department of General, Visceral and Vascular Surgery, University Hospital Jena; the analysis overlay was provided by André Homeyer, Fraunhofer MEVIS, Bremen.

Two of these length scales of inhomogeneity are most relevant for metabolic processes. On the sinusoidal length scale, effects occurring in specific regions along sinusoids are denoted by *zonation*[12, 13]. In case we do not refer to a small number of zones, we will denote this by *sinusoid-scale heterogeneity*. Furthermore, metabolic effects can differ between lobuli found at different locations in or across lobes. We will denote the latter by *organ-scale heterogeneity*. Generally, three main reasons for inhomogeneous metabolization can be distinguished: *(a)* The *periportal* hepatocytes, those near the inflow to the sinusoid, experience a higher concentration of a compound being metabolized than the *pericentral* hepatocytes, those near the outflow. This may lead to concentration gradients even if the cells themselves do not differ in terms of their metabolic capability. *(b)* Different gene expression or enzyme levels depending on the location, either along sinusoids or throughout the organ, may additionally lead to spatial differences in the metabolic capability. *(c)* Pathological states can furthermore affect the metabolic capability, see the examples in Fig 1 showing steatosis patterns at different length scales.

The goal of this article is to introduce a generic simulation framework capable of dealing with both types of spatial inhomogeneity, i.e., sinusoid-scale as well as organ-scale heterogeneity. It is intended to mechanistically model the underlying effects and allow predictions in the case of pathophysiological changes. The key building block of the multiscale model presented here corresponds to the sinusoidal scale and is denoted by *representative sinusoids*. Each such representative sinusoid contains a spatial pattern of metabolization parameters and stands for one region of the liver exhibiting essentially this same parameter pattern. An organ-scale heterogeneity can thus be represented in the model by taking into account multiple representative sinusoids in parallel.

### Review of Related Pharmacokinetics Simulation Techniques

Physiologically based pharmacokinetic (PBPK) modeling mechanistically represents whole-body physiology at an organism level. In contrast to rather generic compartments in classical PK modeling, PBPK models consider organs explicitly, allowing amongst others for the simulation of time-concentration profiles in specific tissues. PBPK models may comprise systems of ordinary differential equations (ODE system) in several hundreds of variables and an equally high number of model parameters. The number of independent model parameters, however, is significantly smaller, i.e., around 5 to 10, due to the large degree of prior information contained in the PBPK software tools. For example, physiological model parameters such as organ volumes or organ surface areas are usually available from integrated, species-specific data collections [15]. To calculate the remaining model parameters, only physicochemical properties of a compound, such as lipophilicity or molecular weight, need to be provided by the user to parametrize the PBPK model [15].

For PBPK model building itself, it is essential to have prior information about the governing physiological processes underlying absorption, distribution, metabolization, and excretion. The physicochemical properties of a compound are used to estimate, e.g., tissue permeation by passive distribution. Active processes such as enzyme-catalyzed metabolization or transporter-mediated uptake or secretion may also be mechanistically considered at the organism level by using tissue-specific gene expression profiles [16]. The degree of biological knowledge included in PBPK models hence ranges from tissue-specific gene expression profiles at the cellular scale to anthropometric parameters at the organism level. For model establishment and model validation, time-concentration profiles are required. PBPK is nowadays routinely used in pharmaceutical development specifically supporting the various stages in the research and development process for example for risk assessment [17], pediatric scaling [18] and cross-species extrapolation [19].

#### Sinusoid-Scale and Organ-Scale Homogeneous Models

Models without spatial resolution are frequently used if a zonation of metabolic capability is unknown or does not play a role, e.g., if a compound is not metabolized at all by a given organ. Such models are structured as compartmental models without spatial resolution of organs [20, 21], or combine multiple organs in one generic compartment [22, 23], and therefore assume that a description corresponding to a *well-stirred* setting is appropriate.

#### Sinusoid-Scale Heterogeneous, Organ-Scale Homogeneous Models

Any metabolic process along a sinusoid introduces a spatial concentration gradient, particularly prominent for compounds subject to significant first-pass elimination [24]. Additionally, metabolic capabilities of neighboring cells along a sinusoid can be distributed inhomogeneously along the sinusoid. This can be due to gene/enzyme expression [25, 26] or zonal presence of other cell types [25]. Examples in this category are carbohydrate metabolism [25], ammonia detoxification [27], and cytochrome metabolism for which two cases are considered in this article. Zonated metabolization can also be caused by pathological conditions appearing zonally, two examples are considered in this article: necrosis after CCl_4_ intoxication [28] and different states of steatosis [29]. Processes involving sinusoid-scale heterogeneity can be modeled by using sequential well-stirred compartments [4, 5, 30, 31] for the liver. Other approaches build a zonation into lobule-scale models containing multiple sinusoids and their surrounding cells (cf. [32] modeling lobular perfusion and [33, 34] modeling lobular perfusion and PK) or into multiphase continuum models (cf. [35, 36] modeling lobular perfusion and [37] modeling lobular perfusion and PK).

#### Organ-Scale Heterogeneity

Metabolization may additionally be heterogeneous at the organ scale, due to differences between sinusoids at different position in the organ. This is mainly due to pathological conditions varying at this length scale. Examples include steatosis [29, 38], fibrosis due to nonalcoholic steatohepatitis [39] or in case of hepatitis C [40], cirrhosis [41], granuloma [41], and carcinoma [42, 43]. Another reason can be heterogeneous concentration sources, e.g., due to intrahepatic injection via catheters [44] or targeted drug delivery [45]. Appropriate modeling techniques for the case of organ-scale heterogeneity without additional zonation are given by multiple parallel well-stirred compartments per organ [20, 31, 46, 47]. Organ-scale porous-medium-based multiphase continuum models [8] can be used as well. These are, however, of limited practical applicability if also a sinusoid-scale heterogeneity is present as they require high spatial resolution in this case. A combination of multiple parallel and sequential well-stirred compartments [31] per organ can be used in case of organ-scale heterogeneity combined with zonation. Also a parallel use of recent cell-based [34] or multiphase porous-medium-based [37] lobule-scale models is conceivable.

In any case, the structure of the model should be chosen depending on what is relevant for the application being considered. It should sufficiently detailed to capture relevant effects, but no more detailed to avoid unnecessary need for parameters and potentially error-prone model assumptions, and to keep computational workload at bay. This in particular implies that it can be useful to consider different degree of detail for representing different organs or organ groups [23].

We point out that this section not meant to be an exhaustive literature review of PK modeling. For more detailed reviews, we refer to [4, 5, 31].

### Introducing a Multiscale Simulation Framework Based on Representative Sinusoids

Our concept of modeling the liver as a parallel connection of representative sinusoids, each of which has cells aligned serially, is based on ideas going back to [20, 30, 47, 48]. The advancement proposed here is threefold: *(a)* By embedding the representative sinusoids in a multiscale simulation framework that separates the scales, all relevant hierarchies can be taken into account efficiently. *(b)* On the organ scale, our simulation is capable of using individualized organ geometries and pathology parameters. *(c)* On the sinusoidal length scale, we model the blood flow by advection along a sinusoid rather than a serial flow through well-stirred compartments. This permits a more accurate temporal resolution at second or sub-second time scales and represents the blood flow as a process in its own right. For a more detailed overview on fluid transport systems in different organs, we refer to [49]. We here use mathematical advection and reaction equations to model blood flow through blood vessels, trans-vascular compound exchange, and chemical kinetics. A more detailed description of the biological processes we model here is given in [50].

The approach presented here is also an extension of our previous work [8] in which the organ is resolved at a single scale as a porous medium. We here consider two spatial scales for the organ tissue: representative sinusoids and individual cells. One such representative sinusoid can capture sinusoid-scale heterogeneity at the appropriate physiological length scale, while different representative sinusoids can be assigned to different locations throughout the organ in case of organ-scale heterogeneity. This approach can be embedded in a simulation framework using multiple spatial scales [51]. At the cellular scale, effective behavior from the finer intracellular scale is integrated in the model via the parameters in the exchange/metabolization ODE system. For the coarser organism scale, our technique can act as a ‘liver module’. The organism scale is currently implemented by an ODE system representing recirculation through the body allowing for metabolization also in other organs, albeit not at the level of detail as in the liver. Other multiscale PK modeling approaches include, e.g., the integration of intracellular signaling [15] or metabolic networks [52] into the cell representation. We refer to [53, 54] for two overviews on multiscale PK modeling approaches and to [55] for approaches of integrating intestinal models and an effective description of the body scale.

#### Comparison to Related Approaches

Our approach has the same goal of multiscale liver modeling as the two recent models [37] dealing with glucose metabolism and [34] considering ammonia detoxification. These approaches differ on a technical level and thus have slightly different foci. The model in [37] considers homogenized multiphase flow in a 2D model lobulus and determines flow velocities as part of the simulation. The model in [34] uses a 3D slice as part of a lobulus with sinusoids and individual hepatocytes. In addition to performing metabolization, the hepatocytes in [34] can grow, divide, and migrate, which allows modeling regeneration in more detail. Consequently, these approaches provide more detail at the lobular level than the simplified 1D sinusoids used in our approach. This, however, implies a higher computational workload for simulation when using more detailed lobular models. While it is certainly possible to extend these to including organ-scale heterogeneity, i.e., many different lobule models with different parameters, efficiency then becomes a challenge.

On the cellular level, [37] considers model of glucose metabolism obtained by model reduction from a detailed kinetic model, whereas [34] and our approach use equations of a given structure with parameters representing biochemical properties and/or parameters fitted to experimental data. It should, however, be possible to use any of these models or also more detailed intracellular models in either approach. Similarly, the extrahepatic models differ, but could in principle be exchanged between the different approaches. In [37], liver inflow concentration profiles are used as model input, corresponding to an isolated perfused liver [56, 57] model. In [34], a three-compartment body-scale recirculation model is used. We consider an isolated perfused liver in one of the example applications presented below, the other two applications involve a multi-compartment body-scale recirculation model representing each individual organ in the respective human or murine body. Given appropriate model parameters, this permits a more detailed investigation of the role of the different organs for the compound being investigated. Detailed recirculation models increase the computational workload of the simulation. This is not a major challenge for our purpose as the additional workload for the extrahepatic model does not scale with the degree of hepatic organ-scale heterogeneity.

#### Model Structure

**Fig 2:**
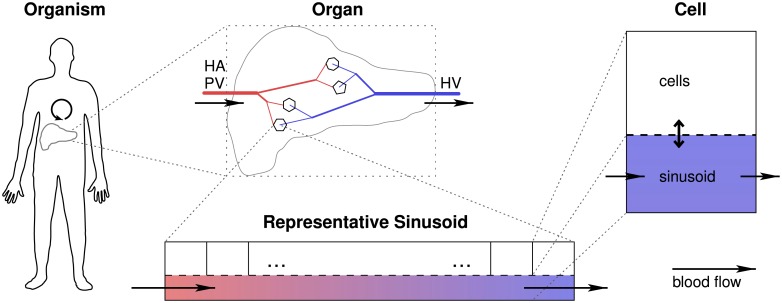
Model Overview. The figure shows the four spatial scales present in our multiscale pharmacokinetics simulation framework. The connection between the scales is given by the blood flow, indicated by arrows. The ‘representative sinusoids’ are the central building blocks in our framework. For each of these, we solve an advection-reaction problem. (HA: hepatic artery; HV: hepatic vein; PV: portal vein.)

Our model consists of four separate building blocks, each representing a distinct physiological spatial scale as described above, see Fig 2. The connection from body via organ and sinusoids to the cells, and back, is provided via the blood flow. Bile flow and lymph flow, such as considered in [36], are not part of our model as the flowing volumes are negligible compared to the blood flow.

Recirculation through the organism *(body scale)* outside the liver as well as possible accumulation and metabolization in other organs are considered in effective form and in particular not at the same degree of detail as for the liver. For this purpose, an ODE system describing the whole-body PBPK model for the substance under consideration is used. These ODE systems are solved numerically using standard backwards differentiation formula (BDF) techniques [58]. The recirculation takes into account the temporal delay of the blood flowing through the organism.

On the *organ scale*, the heterogeneity of metabolization or pathology parameters determines the number and size of regions of different parameter values needed. To represent the connection between the organism and the sinusoids, the hepatic vascular systems distributing the blood throughout the organ are taken into account. The concentrations flowing from the body to the liver are hereby transported to each representative sinusoid with a given temporal delay. The outputs of the representative sinusoids with the respective temporal delays are summed up and form the concentrations flowing from the liver back to the body. In both vascular systems, only advection is considered. In particular, exchange between red blood cells and plasma and any metabolization processes are omitted in the blood vessels. As velocities are constant along each edge and over time, an advection simulation as in [8] is not necessary here and it is sufficient to consider appropriate delay times for different paths through the vascular system. If the actual geometrical configuration of regions of the same sinusoid-scale parameter patterns does not need to be reflected in the simulation, in particular if no exact temporal correspondence between different representative sinusoids is necessary, the vascular systems do not need to be considered for the simulation. In this case, only the relative contribution of each representative sinusoid for the whole organ and possibly the respective temporal flow delay are needed. Let us, however, point out that the simulation results can still be mapped to the actual geometric position for interpretation or visualization if needed. Depending on the organ-scale parameter heterogeneity, the liver model contains several representative sinusoids.

The representative sinusoids *(sinusoidal scale)* are used to model the actual exchange of compounds between the blood and the liver cells as well as the metabolization in the cells. For this purpose, the sinusoid is viewed as consisting of four subspaces: red blood cells (RBC), plasma, interstitium, and cells. The interstitium mainly represents the physiological space of Disse, the cells primarily refer to the hepatocytes. This means that the actual sinusoid only corresponds to the red blood cells and the plasma. However, to simplify terminology, we view the adjacent interstitium and cells as part of the representative sinusoid. The compound under consideration is exchanged between the subspaces and metabolized in the cellular subspace. This structure is chosen in analogy to the liver subcompartments in PBPK models [59] and was previously used in [8]. In addition, the compound concentration is subject to blood flow with constant flow velocity in the RBC and plasma subspaces. Exchange and metabolization at the *cellular scale* are described by cell-specific ODE systems with potentially different parameters to reflect sinusoid-scale heterogeneous patterns. Blood flow is represented by a one-dimensional advection PDE, so that we obtain an advection-reaction problem for each representative sinusoid. We will apply a combination of a Eulerian–Lagrangian Localized Adjoint Method (ELLAM) [60] and Runge–Kutta–Fehlberg 4th/5th order [61] time stepping to solve these numerically. Our approach considers sinusoids at their correct physiological length scale, even though one sinusoid in the model represents larger regions in the liver. In contrast to models using a series of well-stirred compartments without delay [20, 30, 47], modeling the blood flow by advection inherently represents the physiological delay of flowing through a sinusoid with a given velocity. This provides more accurate temporal resolution at sub-second or second time scales, which is a relevant time scale for some applications, e.g., [62].

### Outline

The present article is further organized as follows: We introduce our multiscale model starting from a PBPK model; describe the processes included at additional scales; how this is modeled, discretized, and implemented; and how two pathological cases are modeled. Three example applications illustrate that this simulation approach is applicable to a broad range of pharmacokinetic and physiological questions. We consider *(a)* the clearance of midazolam in human livers affected by steatosis, a zonated process subject to a zonated and organ-scale heterogeneous pathological condition, *(b)* the clearance of caffeine in regenerating mouse livers, a zonated process subject to a zonated pathological condition changing over time, and *(c)* a parameter study on different cell entities performing insulin uptake in mouse livers, investigating different spatial patterns of the cell entities along a single sinusoid. Finally, we discuss the present limitations and further perspectives.

## Methods

In this section, we describe the components of our multiscale model, i.e., the building blocks at the different spatial scales. We start addressing the body and cellular scale where we use and extend an existing PBPK model using well-stirred compartments for each organ. Next, geometry and flow on the organ scale is considered before we address the sinusoidal scale, introducing the key component of our approach. The interaction of the building blocks and necessary translations for models of different dimensionality are presented in. Finally, we introduce two model perturbations representing diseased states of the liver. An overview of the models and their dimensionality for the processes at the different spatial scales is given in Table 1. Specific compounds and their PBPK models for the cellular scale are addressed later in the results section.

**Table 1:** Processes and Numerical Techniques. The table gives an overview of the processes being modeled and the numerical techniques applied in each of the model building blocks for the distinct spatial scales, cf. Fig 2. The overall model requires an integration of submodels of different dimensionality: from 0D, referring to models where spatial effects are not explicitly considered, via 1D sinusoids to a 3D organ. Round brackets in the table refer to equations in this article, square brackets to literature.

Scale	Process(es)	Mathematical Model and Discretization	Equation(s)
**Organism**	recirculation	0D ODE system BDF (CVODE) [58]	
**Organ**	distribution by perfusion	0D temporal delay, averaging evaluated directly	Eqs 7, 8
		3D geometry: Fig 3	
**Sinusoid**	blood flow and cellular uptake	1D advection–reaction PDE: advection: ELLAM [60]	Eq 9
**Cell**	exchange and metabolization	0D ODE system: reaction: RKF45 [61]	Eqs 9, 4, 15

The presented methodology is not limited in the number of compounds. Nevertheless, we restrict the presentation to the case of a single compound throughout this section. At the respective positions, we briefly address how the methods can be extended to multiple compounds, in particular when taking into account the formation of metabolites.

### Model Building Blocks at the Body and Cellular Scales

The starting point for our multiscale model is a whole-body PBPK model implemented in PK-Sim [59] where organs are represented as well-stirred compartments, i.e., zero dimensional. This model without its liver component will be adapted for simulating recirculation through the organism, the liver component will be used as the basis of our refined liver model. Let us point out that the body-scale recirculation can also be omitted if an isolated perfused liver [56, 57] is considered.

#### Body-Scale Recirculation Model

The body-scale PBPK model [59] describes the blood flow between the organs, and thus the transport of a compound through the body, as well as the exchange of compounds between subcompartments of the organs and the metabolization. These processes are described by a non-linear ODE system in several hundred variables.

On the body scale, it is necessary to consider a temporal delay of the recirculation. In certain cases, more than one initial pass of an injected compound can be observed experimentally, see, e.g., [62] for measurements of antipyrine in dogs or [63] for an MRI contrast agent in humans. An ODE model not taking into account the temporal delay of the blood flow between and through the different organs is generally not capable of reproducing second and further passes. This is not an issue for observations on the time scale of hours or days, but should not be ignored when simulating processes on the time scale of seconds to minutes.

In our model, we integrate this temporal delay of the recirculation as a delay *τ*_body_ for blood flowing between the lung and the systemic arterial blood pool, the latter supplying all organs in the model. This delay thus affects the compound mass flows in the red blood cells and the plasma from the lung to the arterial blood pool. This location in the PBPK model was chosen because all recirculating blood passes through the lung. The body delay *τ*_body_ was computed as follows: First, let *τ*_rec total_ denote the total recirculation time through the body obtained as total blood volume in the organism divided by the blood flow rate through the lung [59], resulting in

$$\tau_{\text{rec}\;\text{total}}=\{\begin{array}{cc} 54.8\;\text{s} & \text{in}\;\text{humans,}\\ 19.7\;\text{s} & \text{in}\;\text{mice.} \end{array}$$ (1)

We refer to the results section below for a discussion about how these values differ from the measurements in [63]. The recirculation time [64] and time between peaks can be expected to differ due to intra-organ retention of compounds. From the values *τ*_rec total_, two transit times explained below are subtracted to obtain *τ*_body_, namely the sinusoidal transit time, see Eq 6, and the transit time for the hepatic vascular systems, if present in the model, see Eqs 7 and 8.

Clearly, this recirculation delay model is not mechanistic, highly simplified, and does not take into account flow delays within or between the individual organs. In a similar manner as for the whole body, we could use specific transit times for each organ, namely dividing the respective blood volume by the respective flow rate. This would make the implementation more technical and increase computational costs for the simulations, but it is not clear to what extent the overall model accuracy would benefit. As this article focuses on the liver, we will leave the recirculation delay model open for future model refinement.

The ODE systems describing recirculation turned out to be very stiff. For solving stiff ODE systems, various numerical methods are available [65, 66]. Based on a brief performance evaluation, we chose a scheme using a BDF scheme available as part of CVODE solvers [58], based on [67], in the SUNDIALS library [68] to solve the recirculation ODE systems. This part of the simulation turned out not to be very time critical, so passing values between our simulation and an external ODE solver is no performance bottleneck.

#### Physiologically Based Pharmacokinetics Models Used for the Cellular Scale

The recirculation ODE systems are expressed in terms of the molar amounts of the compound in different organs. For introducing spatial resolution, we use molar concentrations in the other building blocks of our multiscale model and as the interface to the recirculation model. Throughout this article, concentration is always meant as molar concentration in units mM = mol m^−3^, unless stated otherwise.

To describe the connection between organism and organ, we generally distinguish between concentrations *c*_f_ flowing in the blood and concentrations *c*_s_ in the surrounding tissue. Moreover, let $c_{\text{f}}^{\text{BL}}(t)$ denote the liver inflow concentration (‘body to liver’). We can then describe well-stirred flow, inter-subcompartmental exchange and intracellular metabolization in terms of compound concentrations as

$$d_{t}\;[\begin{array}{c} c_{\text{f}}\\ c_{\text{s}} \end{array}]=\alpha \;[\begin{array}{c} c_{\text{f}}^{\text{BL}}-c_{\text{f}}\\ 0 \end{array}]+E([\begin{array}{c} c_{\text{f}}\\ c_{\text{s}} \end{array}])+\;[\begin{array}{c} 0\\ m(c_{\text{s}}) \end{array}]$$ (2)

where we omitted the dependency on time for a more concise notation. The flow rate *α* > 0 indicates which fraction of the blood volume in the organ is exchanged per time unit. The function *E* describes the exchange between the subspaces in the representative sinusoid, and *m* the metabolization. For multiple compounds, the exchange terms *E* typically apply separately for each compound. In contrast, the metabolization term *m* can model metabolic conversion of compounds to the respective metabolites and thus involve concentrations of multiple compounds.

Throughout this section, we will consider a model structure consisting of red blood cells and blood plasma as subcompartments subject to flow. Interstitium and cells will be considered as stationary subcompartments. The corresponding molar concentrations of the compound being considered are denoted by *c*_rbc_, *c*_pls_, *c*_int_, and *c*_cel_, respectively. In this setting, the cellular subcompartment summarizes the contribution of all cells to the exchange and metabolization of the compound. In the liver, the cellular subcompartment mainly represents hepatocytes, spatial patterns of which we focus on throughout this article.

This model structure applies to two of the applications below, midazolam and caffeine, and serves as the example for the presentation of the methods. If only passive, gradient-driven exchange of compounds takes place, we can write *E* as the multiplication by a 4 × 4 matrix of the form

$$\left[\begin{array}{cccc} \frac{-P_{\text{rbc,pls}}}{\kappa_{\text{rbc,pls}}\varphi_{\text{rbc}}} & \frac{+P_{\text{rbc,pls}}}{\varphi_{\text{rbc}}} & 0 & 0\\ \frac{+P_{\text{rbc,pls}}}{\kappa_{\text{rbc,pls}}\varphi_{\text{pls}}} & \frac{-P_{\text{rbc,pls}}}{\varphi_{\text{pls}}}+\frac{-P_{\text{pls,int}}}{\varphi_{\text{pls}}} & \frac{+P_{\text{pls,int}}}{\kappa_{\text{pls,int}}\varphi_{\text{pls}}} & 0\\ 0 & \frac{+P_{\text{pls,int}}}{\varphi_{\text{int}}} & \frac{-P_{\text{pls,int}}}{\kappa_{\text{pls,int}}\varphi_{\text{int}}}+\frac{-P_{\text{int,cell}}\kappa_{\text{int}}}{\varphi_{\text{int}}} & \frac{+P_{\text{int,cell}}\kappa_{\text{cell}}}{\varphi_{\text{int}}}\\ 0 & 0 & \frac{+P_{\text{int,cell}}\kappa_{\text{int}}}{\varphi_{\text{cell}}} & \frac{-P_{\text{int,cell}}\kappa_{\text{cell}}}{\varphi_{\text{cell}}} \end{array}\right]$$ (3)

as used in own earlier work [8]. Here, *φ*_{rbc, pls, int, cell}_ are the volume fractions specified below in Eq 5, *κ*_rbc,pls_, *κ*_pls,int_, *κ*_int_, and *κ*_cell_ are dimensionless partition coefficients describing the equilibrium state of molar concentrations at which the respective individual exchanges vanish, *P*_rbc,pls_, *P*_pls,int_, and *P*_int,cell_ are the local effective permeabilities [s^−1^] between the different subspaces of the representative sinusoid. The metabolization *m* is, in our case, described by Michaelis–Menten kinetics [69] of the form [8]

$$m(c_{\text{cell}})=-\frac{V_{\text{max}}^{\text{cell}}\kappa_{\text{cell}}c_{\text{cell}}}{K_{m}^{\text{cell}}+\kappa_{\text{cell}}c_{\text{cell}}}$$ (4)

with parameters $V_{\text{max}}^{\text{cell}}$ describing the maximum metabolization rate [s^−1^] and $K_{m}^{\text{cell}}$ describing the molar concentration [mM] at which half $V_{\text{max}}^{\text{cell}}$ is attained. In this form, only the removal of a single compound from our system is represented. More complex functions *E* and *m* are required if several compounds are considered, as demonstrated in the insulin example in the results section.

The volume fractions *φ*_{rbc,pls,int,cell}_ are specific for different species, in our case the volume fractions for humans [59] are

$$\varphi_{\text{rbc}}=0.077,\;\varphi_{\text{pls}}=0.093,\;\varphi_{\text{int}}=0.163,\;\varphi_{\text{cell}}=0.667.$$ (5a)

where as volume fractions for mice [59] are

$$\varphi_{\text{rbc}}=0.052,\;\varphi_{\text{pls}}\;=0.063,\;\varphi_{\text{int}}=0.163,\;\varphi_{\text{cell}}=0.722,$$ (5b)

The exchange and metabolization parameters *P*_⋆_, *κ*_⋆_, $V_{\text{max}}^{\text{cell}}$, and $K_{m}^{\text{cell}}$ depend on compound and species and are given below for our example applications.

The typical transit time *τ*_typ_ for the sinusoidal scale is obtained as the ratio of the sinusoidal volume fraction *φ*_sin_ = *φ*_rbc_ + *φ*_pls_ over the total liver blood flow, both values taken from [59]. Thus, *τ*_typ_ satisfies the relation *α* = 1/*τ*_typ_ for the flow rate *α* from Eq 2, its value is

τtyp={14.3sinhumans,13.6sinmice. (6)

For a discussion of the precise form of the flow, we refer to Eq 9 and its description. We are aware that this transit time is not in good agreement with experimental data for sinusoidal flow velocities for humans [70] or rats [71, 72], using lobular diameters [1, 7] as a lower bound on sinusoidal length. Due to the large variability of the experimental velocity measurements, we decided using the values in Eq 6 for *τ*_typ_ consistent with the underlying whole-body models [59]. These values can, however, easily be exchanged if a better estimate is available.

### Organ-Scale Model Building Blocks

We will consider a spatially resolved extension of Eq 2 for cells organized along sinusoids as described below. For being able to describe the extracellular concentrations, inflow values for the sinusoids need to be obtained from the body scale and the sinusoidal outflow needs to be passed back to the body scale. For this purpose, a realistic 3D geometric model of the hepatic vascular systems is used. In these, we model piecewise 1D blood flow.

#### Blood Flow Model

For simplicity, we only consider a single supplying vascular system denoted by SV, comprising the physiologically present HA and PV, and already compute the mixed concentration of arterial and portovenous blood at the liver inflow. This is clearly a simplification and would need to be modified if we wanted to consider locally varying relative contributions of the two blood types [73, 74]—for which, however, we would need appropriate data. As another simplification, we assume constant flow velocities across vascular cross sections satisfying Poiseuille’s law [75], which reduces our flow model to flow on a branching one-dimensional domain. The Fåhræus–Lindqvist effect [76], the decrease of the effective viscosity of blood in blood vessels of radius ≤ 150 μm, is accounted for in the flow velocities. As we do not focus on the local flow in the vascular structures, we do not consider the precise velocity profile across cross sections or near bifurcations. Moreover, we assume that no metabolization and no exchange between plasma and red blood cells takes place in the vascular systems. This simplifies the model so that we only need to take into account advection by blood flow for which explicit formulas can be given. For this purpose, concentrations in the plasma and the red blood cells as well for all compounds present in the blood flow can be treated in the same way as explained next, and we omit the respective indices for notational convenience.

**Model:** For a given point *x* in the supplying vascular system, we can compute a temporal delay *τ* ^SV^(*x*) between the inflow and *x*, so the concentration $c_{\text{f}}^{\text{SV}}(x,t)$ in the SV at position *x* and at time *t* can easily be computed from the liver inflow concentration $c_{\text{f}}^{\text{BL}}(t)$ as

$$c_{\text{f}}^{\text{SV}}\left(x,t\right)=c_{\text{f}}^{\text{BL}}(t-\tau^{\text{SV}}\left(x\right))\;.$$ (7)

This allows computing the inflow concentrations for all representative sinusoids by substituting the respective connection point for *x*.

For a point *x* in the draining vascular system, this is slightly more technical since the flow from multiple representative sinusoids contributes to the concentration at *x*, each with potentially different delay. Let *R*(*x*) denote all these representative sinusoids and *τ* ^HV^(*r*, *x*) the temporal delays for all *r* ∈ *R*(*x*). Moreover, let *w*_*r*, *x*_ denote the relative contributions of the flows to the flow at *x*, determined from flow velocities and cross section areas of the respective edges. Then the concentration $c_{\text{f}}^{\text{HV}}(x,t)$ in the HV at position *x* and at time *t* is given as a weighted, delayed sum of outflow concentrations $c_{\text{f}}^{r,out}$ of representative sinusoids *r*, namely

$$c_{\text{f}}^{\text{HV}}\left(x,t\right)=\sum_{r\in R(x)}w_{r,x}\cdot c_{\text{f}}^{r,\text{out}}(t-\tau^{\text{HV}}\left(r,x\right))$$ (8)

to obtain the concentration at point *x* and time *t*. The liver outflow concentration *c*_BL_ (‘liver to body’) is then easily obtained by evaluating Eq 8 at the root of the HV.

**Implementation:** The evaluation of Eqs 7 and 8 does not require sophisticated simulation techniques. The approach merely makes it necessary to store concentration history at the transfer points, but at no other locations. Thus, a spatial discretization of the concentrations in the vascular systems is not necessary. Storage is only needed for a limited period of time, long enough so that appropriate interpolation is possible for the maximal transition time, which limits memory consumption. In contrast, spatial concentration profiles for the vascular trees as well as data for advection simulation is not necessary at all, saving memory and computation time compared to a full-featured advection simulation for the vascular systems as performed in [8]. Discrete time points are given by the outer simulation loop and may require piecewise first-order polynomial interpolation of concentrations in time.

#### Obtaining Realistic Geometric Models of Organ and Vasculature

**Fig 3:**
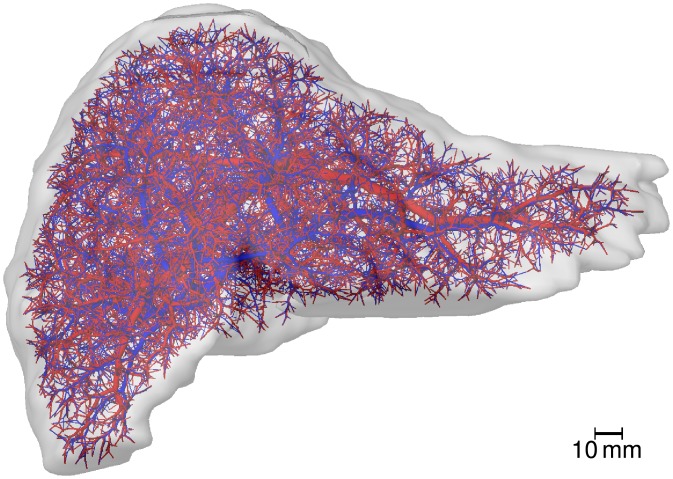
Human Liver Vascular Dataset. The image shows a visualization of a human liver shape including algorithmically refined vascular structures. The supplying vascular system, comprising portal vein and hepatic artery, is shown in *red*, the draining vascular system (hepatic vein) in *blue*.

A realistic 3D geometric model of a human liver and its vascular structures was obtained by applying the same workflow described in [77] for in vivo μCT scans of mouse livers. In summary, an abdominal in vivo MRI scan of a healthy 31-year old male Caucasian was used to segment the liver and its vascular structures [78]. The latter were subsequently skeletonized and converted to a graph representation using a semi-automatic procedure [78]. The vascular graphs were simplified to strictly bifurcative trees with cylindrical edges as described in [79]. They were refined algorithmically [80] to the desired degree of detail as described in [79]. For this purpose, the same set of 10 000 end points for the supplying and draining vascular systems were used. Each of these points represents groups of about 5^3^ physical lobuli, leading to a total of 1.25 million lobuli, a realistic number [1]. In each group, we assume the same metabolic properties for all sinusoids. The underlying organ mask an the resulting vascular system are visualized in Fig 3 and provided as S1 Dataset.

For a fully lobular resolution, one would have to take into account how the blood supplied by one portal field is distributed to multiple surrounding central veins, an effect we neglect in the approach here. We could, however, easily incorporate it for applications where such a more detailed draining pattern is of importance.

Our applications involving mouse models presented here did not require 3D geometric models as these examples did not include organ-scale heterogeneity. Such geometric models are available as supporting information to [8] and [77] and could easily be included in the simulations if organ-scale heterogeneity needs to be included.

### Sinusoid-Scale Model Building Blocks

The sinusoidal scale is the central building block for our multiscale modeling framework. The processes modeled here, in 1D, are relatively simple, but a suitable adaption of parameters, as described in the model integration below, will still be necessary.

#### Sinusoidal Blood Flow and Pharmacokinetics Model

For the sinusoidal scale, we again start with the general model form as in Eq 2 distinguishing flowing and stationary concentration. The 1D advection-reaction for one representative sinusoid can then be expressed as

$$\partial_{t}[\;\begin{array}{c} c_{\text{f}}\\ c_{\text{s}} \end{array}]+[\begin{array}{c} v\partial_{x}c_{\text{f}}\\ 0 \end{array}]=E\;([\begin{array}{c} c_{\text{f}}\\ c_{\text{s}} \end{array}])+[\begin{array}{c} 0\\ m(c_{\text{s}}) \end{array}]$$ (9)

where *E* and *m*, as above, denote the exchange of concentrations between the subspaces and the metabolization, respectively, and *v* is the one-dimensional flow velocity. Boundary values at the inflow are those provided by the supplying vascular system, outflowing values at the other end of the sinusoid are computed and passed to the draining vascular system. In the current model version, we in particular neglect diffusion outside the cells, in particular in the space of Disse, as well as exchange between neighboring cells due to gap junctions [81].

For the reaction part of Eq 9, multiple compounds may play a role. On the one hand, one compound may change the exchange or metabolization behavior for another compound if the underlying bio-chemical processes involve such an interdependence. On the other hand, metabolization itself can produce metabolites (i.e., other compounds) from compounds.

Clearly, the blood flow through sinusoids is probably better described as an intermittent, corpuscular flow involving a non-Newtonian fluid through sinusoids of complex geometry that is not much larger than the red blood cells. We refer to Video S1 of [82] for an illustration of this blood flow. However, for a representation of the average flow behavior, as intended here, we believe this strong simplification to constant flow velocity to be an appropriate description.

#### Discretization and Implementation

As in [8], we discretize the advection and reaction parts of Eq 9 separately. This avoids a rather technical implementation of a numerical scheme capable of treating both processes simultaneously, even though this is possible even for a nonlinear reaction term [83]. The simulation moreover does not require a small time step appropriate for both processes, but needs appropriate time steps for the two processes separately and an appropriate synchronization strategy.

We do not resolve the internal spatial structure of hepatocytes and thus assume them to be well-stirred. It is thus a natural choice to use a resolution of one grid point per hepatocyte for the spatial discretization of the representative sinusoids. This is also the spatial resolution at which sinusoid-scale parameter heterogeneity needs to be given.

**Advection:** While pure 1D advection problems with constant velocity can be solved analytically, this is no longer possible if a reaction term is present as well, so it needs to be addressed numerically. The simplest implementation of time stepping for the advection part of Eq 9 could be obtained by choosing the time step such that the velocity is one grid cell per time step. This, however, would not allow joint time steps if velocities in different representative sinusoids differ, and might be too large a time step for synchronizing with fast reactions, so we need a numerical advection scheme that can deal with arbitrary velocities. An ideal such discretization scheme would conserve mass and neither introduce numerical diffusion nor create spurious oscillations [84]. However, state-of-the-art methods do not simultaneously satisfy all three properties, so a compromise is needed. For our purposes, we particularly focus on mass conservation and avoiding spurious oscillations, which might result in overshoots and negative concentrations, so we need to accept numerical diffusion effects.

We use a 1D Eulerian-Lagrangian Localized Adjoint Method (ELLAM) [60], going back to [85] with more details on mathematical analysis in [86]. This method was preferred over other discretization schemes for advection [87] as it prevents numerical artifacts [88]. To prevent artificial oscillations, mass lumping as suggested in [89, 90] was used. While ELLAM could, in principle, treat more than advection simultaneously [83, 91, 92], also for non-linear reactions [93], we treat flow, exchange/metabolization, and recirculation separately by appropriate methods. The scheme was implemented in custom C++ code based on own earlier work, Section 1 in the supporting information Text S1 of [8] and [94]. In summary, the ELLAM scheme for advection amounts to solving a linear system of equations to update the concentrations for the compounds present in the blood flow in each time step.

**Reaction** For the reaction part of Eq 9, we observed no issues due to stiffness of the ODE systems. So we chose a standard Runge-Kutta-Fehlberg 4th/5th order (RKF45) [61] scheme that automatically adapts the time step size. This method is recommended as a ‘good general-purpose integrator’ in the GNU Scientific Library [95]. The RKF45 performed well in this case, so we do not need to apply more sophisticated methods as recommended in [96, 97]. We used a custom C++ implementation of an RKF45 scheme from [8] to avoid data exchange with external libraries as these ODE solution steps are rather time-critical in the simulation.

### Model Integration in Representative Sinusoids

Besides a connection of the models on the distinct scales, an adaption of parameters due to the different dimensionality of the models is necessary. This affects the volume fractions, the respective surface areas and thus the effective permeabilities, and the length of regions along the sinusoid. Moreover, we discuss an additional model fitting step needed if our cellular model was not parametrized for the model structure involving representative sinusoids.

#### One-Dimensional Representation of Sinusoids

To account for increasing sinusoid diameter from portal fields to central veins in a 1D representation of the blood flow in 3D lobuli, we need to find an appropriate representation. As variations of the processes in longitudinal direction of the lobulus are of minor importance, we can restrict our view to 2D cross sections of lobuli. Mathematically, the goal of this subsection is to describe how parameters of the PBPK liver submodel need to be adapted. This adaption will involve scaling some of the parameters and replacing the constant volume fractions *φ*_*i*_, *i* ∈ {rbc, pls, int, cell}, by volume fractions *ψ*_*i*_(*λ*) depending on the position *λ* along the sinusoid.

For this purpose, our approach is to consider one physiological sinusoid starting from the portal field. As it combines with other sinusoids towards the central vein, the blood flow from the one, portally starting, sinusoid is only a certain fraction between 0 and 1 of the blood flow through the centrally ending sinusoid. For the lack of more detailed data, we assume that the flow velocity does not change along the physiological sinusoids. Moreover, we assume that the physiological sinusoids are surrounded by an interstitial and a cellular layer of constant width, independent of the sinusoid radius.

Radii of periportal and pericentral sinusoids in mice are *r*_sin,pp_ = 4.4 μm and *r*_sin,pc_ = 6.85 μm [98], respectively. This range is similar to sinusoidal diameters of 7 and 15 μm reported in [1], so we will use the following formulas for both humans and mice. Assuming a linear increase of the cross section area, the sinusoidal radius can be computed as

$$A_{⌀\text{,sin}}\left(\lambda \right)=A_{⌀\text{,sin,pp}}+\lambda (A_{⌀\text{,sin,pc}}-A_{⌀\text{,sin,pp}})=\pi r_{\text{sin,pp}}^{2}+\lambda (\pi r_{\text{sin,pc}}^{2}-\pi r_{\text{sin,pp}}^{2})$$ (10a)

$$r_{\text{sin}}(\lambda )\;=\sqrt{\frac{A_{\emptyset \text{,sin}}(\lambda )}{\pi }}=\sqrt{r_{\text{sin,pp}}^{2}+\lambda \left(r_{\text{sin,pc}}^{2}-r_{\text{sin,pp}}^{2}\right)}$$ (10b)

where *λ* indicates the position along the sinusoid in units of sinusoid length, i.e., *λ* = 0 at the periportal end and *λ* = 1 at the pericentral end.

For the thickness of the interstitium and the representative cellular layer, denoted by *w*_int_ and *w*_cell_, we assume that the volume fractions for mice from Eq 5b correspond to the average sinusoidal cross-section area, i.e., to the radius *r*_sin, avg_ = *r*_sin_(0.5) = 5.757 μm. We then compute

$$w_{\text{int}}=\left(\sqrt{1+\frac{\varphi^{\text{int}}}{\varphi^{\text{sin}}}}-1\right)\cdot r_{\text{sin,avg}}=3.187\;\;\mu \text{m}$$ (11a)

$$w_{\text{cell}}=\left(\sqrt{1+\frac{\varphi^{\text{cell}}+\varphi^{\text{int}}}{\varphi^{\text{sin}}}}-\sqrt{\frac{\varphi^{\text{sin}}+\varphi^{\text{int}}}{\varphi^{\text{sin}}}}\right)\cdot r_{\text{sin,avg}}=8.001\;\;\mu \text{m}$$ (11b)

**Fig 4:**
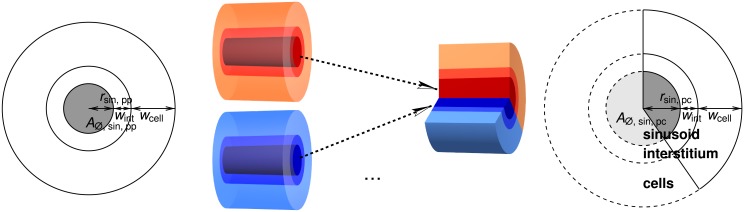
Sketch for the 1D Representation of Sinusoids. The volume rendering in the *middle* illustrates our assumption of multiple periportally starting sinusoids contributing to a thicker pericentrally terminating sinusoid. The width of the interstitial and cellular layer is assumed to remain constant along the sinusoid. The sinusoidal cross-section sketches on the *left* and *right* show that this also has an effect on the respective surface areas, which has an influence on the respective effective permeabilities.

Note that these values refer to volumes representative for the physiological interstitium and cells and are not meant as their precise actual sizes, in particular for the cells. The radii and these thicknesses are illustrated in Fig 4.

Using Eqs 10 and 11, the variation of the volume fractions along the representative sinusoid can be expressed as

$$\psi_{\text{sin}}(\lambda )\;=\frac{\pi {\left[r_{\text{sin}}(\lambda )\right]}^{2}}{\pi {\left[r_{\text{sin}}(\lambda )+w_{\text{int}}+w_{\text{cell}}\right]}^{2}}$$ (12a)

$$\psi_{\text{rbc}}(\lambda )=\frac{\varphi_{\text{rbc}}}{\varphi_{\text{sin}}}\psi_{\text{sin}}(\lambda )$$ (12b)

$$\psi_{\text{pls}}(\lambda )=\frac{\varphi_{\text{pls}}}{\varphi_{\text{sin}}}\psi_{\text{sin}}(\lambda )$$ (12c)

$$\psi_{\text{int}}(\lambda )=\frac{\pi {\left[r_{\text{sin}}(\lambda )+w_{\text{int}}\right]}^{2}-\pi {\left[r_{\text{sin}}(\lambda )\right]}^{2}}{\pi {\left[r_{\text{sin}}(\lambda )+w_{\text{int}}+w_{\text{cell}}\right]}^{2}}$$ (12d)

$$\psi_{\text{cell}}(\lambda )=\frac{\pi {\left[r_{\text{sin}}(\lambda )+w_{\text{int}}+w_{\text{cell}}\right]}^{2}-\pi {\left[r_{\text{sin}}(\lambda )+w_{\text{int}}\right]}^{2}}{\pi {\left[r_{\text{sin}}(\lambda )+w_{\text{int}}+w_{\text{cell}}\right]}^{2}}$$ (12e)

The contribution of the periportally starting sinusoid to the representative sinusoid at position *λ* is given by the ratio of the cross section areas,

$$\rho (\lambda )=\frac{\pi {\left[r_{\text{sin}}(0)\right]}^{2}}{\pi {\left[r_{\text{sin}}(\lambda )\right]}^{2}}=\frac{r_{\text{sin, pp}}^{2}}{{\left[r_{\text{sin}}(\lambda )\right]}^{2}}.$$ (13)

We refer to Fig 4 for a sketch of this interpretation of the contributions. The inverse of this contribution is needed when determining the represented volume by one section of the representative sinusoid. In addition, permeabilities relating to surface areas need to be scaled by a factor derived from *ρ*(*λ*), taking into account the circumference of the representative sinusoid at position *λ* and the fraction of represented there,

$$\sigma \left(\lambda \right)=\frac{2\pi r_{\text{sin}}\left(\lambda \right)}{2\pi r_{\text{sin}}\left(0.5\right)}\cdot \frac{\rho (\lambda )}{\rho (0.5)}\;.$$ (14)

This factor is multiplied by the permeability which we assume to be given for the middle of the sinusoid.

These adaptions imply a modification of the exchange term Eq 3 to

$$E\left(\lambda \right)=[\begin{array}{cccc} \frac{-P_{\text{rbc,pls}}}{\kappa_{\text{rbc,pls}}\psi_{\text{rbc}}\left(\lambda \right)} & \frac{+P_{\text{rbc,pls}}}{\psi_{\text{rbc}}\left(\lambda \right)} & 0 & 0\\ \frac{+P_{\text{rbc,pls}}}{\kappa_{\text{rbc,pls}}\psi_{\text{pls}}\left(\lambda \right)} & \frac{-P_{\text{rbc,pls}}}{\psi_{\text{pls}}\left(\lambda \right)}+\frac{-\sigma \left(\lambda \right)P_{\text{pls,int}}}{\psi_{\text{pls}}\left(\lambda \right)} & \frac{+\sigma \left(\lambda \right)P_{\text{pls,int}}}{\kappa_{\text{pls,int}}\psi_{\text{pls}}\left(\lambda \right)} & 0\\ 0 & \frac{+\sigma \left(\lambda \right)P_{\text{pls,int}}}{\psi_{\text{int}}\left(\lambda \right)} & \frac{-\sigma \left(\lambda \right)P_{\text{pls,int}}}{\kappa_{\text{pls,int}}\psi_{\text{int}}\left(\lambda \right)}+\frac{-\sigma \left(\lambda \right)P_{\text{int,cell}}\kappa_{\text{int}}}{\psi_{\text{int}}\left(\lambda \right)} & \frac{+\sigma \left(\lambda \right)P_{\text{int,cell}}\kappa_{\text{cell}}}{\psi_{\text{int}}\left(\lambda \right)}\\ 0 & 0 & \frac{+\sigma \left(\lambda \right)P_{\text{int,cell}}\kappa_{\text{int}}}{\psi_{\text{cell}}\left(\lambda \right)} & \frac{-\sigma \left(\lambda \right)P_{\text{int,cell}}\kappa_{\text{cell}}}{\psi_{\text{cell}}\left(\lambda \right)} \end{array}]$$ (15)

whereas the metabolization term Eq 4 is unaffected.

#### One-Dimensional Representation of Sinusoid-Scale Heterogeneity

A zonation of metabolic capabilities is typically determined by staining enzymes in histological slices. These images are evaluated by measuring the total cross-section area of different lobuli and the corresponding areas of different staining result. For an enzyme near, e.g., the central veins, the analysis thus allows defining a pericentral area ratio *A*_pc_/*A*_∅_, whereas we need a pericentral length ratio *l*_pc_/*l*_rep. sin._ for the representative sinusoid models. We view lobuli as cylinders around their respective central vein, which is certainly a simplification from 3D to 2D, but a suitable approximation of the actual polygonal shape of lobuli [99]). Then, we can easily convert from central circular area to central length via

$$\frac{A_{\text{pc}}}{A_{\emptyset }}=\frac{\pi l_{\text{pc}}^{2}}{\pi l_{\text{rep.}\;\text{sin.}}^{2}}\;\Rightarrow \frac{l_{\text{pc}}}{l_{\text{rep.}\;\text{sin.}}}=\sqrt{\frac{A_{\text{pc}}}{A_{\emptyset }}}\;.$$ (16)

This conversion also applies when considering the zonation of pathological changes as these can be determined similarly, see, e.g., Fig 1, or, more generally, any sinusoid-scale parameter heterogeneity.

Let us point out that there is no unique ‘periportal’ or ‘pericentral’ zone, they can in particular not necessarily be identified with zones 1 or 3 in the notation of [29, 100]. The size of such zones depends on the process considered. In the caffeine example considered in the results section, there are two pericentral regions: one of fixed size where the enzyme Cyp1a2 is expressed, another one of variable size which is affected by necrosis.

#### From Well-Stirred to Representative Sinusoid Models

Frequently, the PK parameters are fitted for a well-stirred organ-scale model rather than a spatially resolved representative sinusoid model already taking into account the adaptions described by Eqs 15 and 16. In this case, they cannot be used immediately for the cellular scale in representative sinusoids. Our approach is to rescale parameters dominantly causing the differences such that the liver outflow concentrations as model output from the organ-scale homogeneous representative sinusoid model are close to outflow concentrations from the well-stirred organ-scale model. No exact match can be expected here, the sinusoid-scale model is designed to represent the transit time and also exhibits some mixing and other effects due to the advection simulation.

This adaption should clearly be independent of the inflow profile considered. If we were dealing with a linear time-invariant (LTI) system, we could simply determine the impulse responses, i.e., the model output when using a Dirac *δ* impulse as input, of the two models and use these for comparison. The ODE systems considered here, however, are not necessarily linear, so standard methodology for LTI systems (see, e.g., Chapter 2 in [101]) cannot be applied. Instead, we make use of the following pragmatic approach. Assuming that the system behaves approximately linearly for our ranges of input, we consider an inflow profile roughly approximating a *δ* impulse. We then fit a scaling factor for the dominating coefficients in the ODE system so that an appropriate deviation is minimized. We will specify this in the results section for our insulin example. This optimization is performed by iterative interval nesting until a given tolerance is reached.

### Zonated and Organ-Scale Heterogeneous Pathological States of the Liver

We now consider two proofs of concept how pathophysiological changes can be taken into account in our model. Steatosis is an example that can occur zonally, organ-scale heterogeneous form, and the combination thereof. Necrosis after CCl_4_ intoxication occurs in zonal form in a rather homogeneous form across the liver. The damage happens on a time scale of hours and the liver regenerates within days, so we here consider the time dynamics of this pathological state.

#### Simplified Steatosis Model

Steatosis is a common liver disease in humans, it is often caused by alcohol abuse, diabetes, protein malnutrition, obesity or the consequence of other pathological conditions [102]. In steatotic livers, lipids accumulate in the cellular subspace [103]. Most often, this happens pericentrally, but the lipid deposition can also occur in the periportal zone, see [104] and the references therein. In addition, an organ-scale heterogeneity between different sinusoids can be observed [105].

**Model Perturbation:** We represent the effect of steatosis as a change in the equilibrium between cells and interstitium via the respective partition coefficient *κ*_cell, healthy_ = *κ*_cell_ in the PBPK equation Eq 3 in the same manner as presented in [8]: Let Δ*s* be the lipid accumulation due to steatosis, then *κ*_cell_(Δ*s*) is computed from *κ*_cell, healthy_ via

$$\kappa_{\text{cell}}(\Delta s)={\left(\frac{1}{\kappa_{\text{cell, healthy}}}+\frac{10^{\text{log}\;P}-1}{\psi_{\text{cell}}(\lambda )}\cdot \Delta s\right)}^{-1}$$ (17)

where log *P* is the lipophilicity of the compound considered. Clearly, this is a strongly simplified representation of steatosis. It explicitly ignores any other effects of steatosis on the PK parameters such as on metabolic capabilities of the cells [106] and intra-subcompartmental permeabilities [107], any changes in microcirculation [108, 109], organ size (as shown in Table 6 in [110]), and any other effects of steatosis on the organ and the whole organism.

**Synthetic Steatosis Data:** The steatosis data used here are synthetic datasets based on experimental observations from the literature. Besides the healthy case (no steatosis), we consider three types of zonation: predominantly periportal (similar to ‘zone 1’ in the terminology of Fig 1 in [29]), predominantly pericentral (‘zone 3’), and non-zonal (‘panacinar’ or ‘azonal’). For this purpose, we assume a total fat accumulation of 9.2% of the liver volume, corresponding to stage 2 steatosis as observed in [111]. We assign pseudo-random [112] steatosis values Δ*s* to each zone, uniformly distributed in the interval 0.092 ⋅ (ρ(λ))^−1^ ⋅ ζ_z_(λ) ⋅ [(1 − 0.69), (1 + 0.69)] where 0.69 is the coefficient of variation reported in [111], the factor *ρ*(*λ*) from Eq 14 in the denominator cancels when computing the total lipid content, and *ζ*_*z*_(*λ*) controls the zonation via

$$\zeta_{z}\left(\lambda \right)=\{\begin{array}{cc} 1 & \text{non-zonal}\;\text{case}\\ 2-2\lambda & \text{predominantly}\;\text{periportal}\;\text{case}\\ 2\lambda & \text{predominantly}\;\text{pericentral}\;\text{case} \end{array}$$ (18)

**Fig 5:**
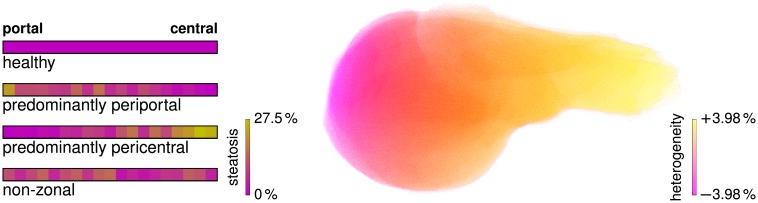
Steatosis Heterogeneity. The *left* images show four examples of different synthetic zonated patterns of steatosis in humans, visualized on a color scale from violet to yellow, corresponding to 0% to 27.5% steatotic lipid accumulation. The three steatotic states correspond to the same total lipid accumulation of 9.2%. The volume rendering on the *right* visualizes the organ-scale gradient *ζ*_*h*_ with a difference of 7.96% lateral direction for a human liver.

Examples for these zonated states of steatosis are visualized in Fig 5.

For introducing an organ-scale heterogeneity, we multiply the ranges above by an additional factor

$$\zeta_{h}\left(x,y,z\right)=1+0.0398\cdot \frac{x-(x_{\text{min}}+x_{\text{max}})/2}{(x_{\text{min}}-x_{\text{max}})/2}$$ (19)

where *x*_min_ and *x*_max_ are the smallest and largest *x* coordinate of the organ. This factor leads to a gradient in the steatosis profile with a difference of 2 ⋅ 3.98% between the leftmost and rightmost point, given by the smallest and largest *x* coordinate, respectively. The value 3.98% is the mean maximum difference between left and right liver as reported in [105] for type-2 diabetic patients, where lower steatosis values are present on the left. A volume rendering in Fig 5 shows this macroscopic lateral steatosis gradient.

#### Simplified Regeneration Model

A frequently used experimental protocol to study toxic liver damage is the administration of CCl_4_ in animals [28]. It induces a necrotic pericentral zone [34], similar to effects of acetaminophen overdoses [113], a frequent cause for acute liver failure in humans [114].

**Model Perturbation:** In a similar manner as in [8], we represent the effect of the necrotic area by replacing the cellular volume by interstitial space, changing the volume fractions in the PK equation Eq 3. As the actual metabolization only takes place in the cellular volume, this effectively prevents metabolization in the necrotic zone. Let us point out that this is again a strongly simplified model. It explicitly ignores any interaction of the CCl_4_ and its metabolites with the compound, any effects of the fragments of the dead cells, any differences in the metabolic capabilities of the regenerating cells to those of the cells natively at the respective position, and any additional influence of the CCl_4_ administration on the whole organism. For a more detailed model of the interplay of regeneration and metabolism we refer to [115].

**Fig 6:**
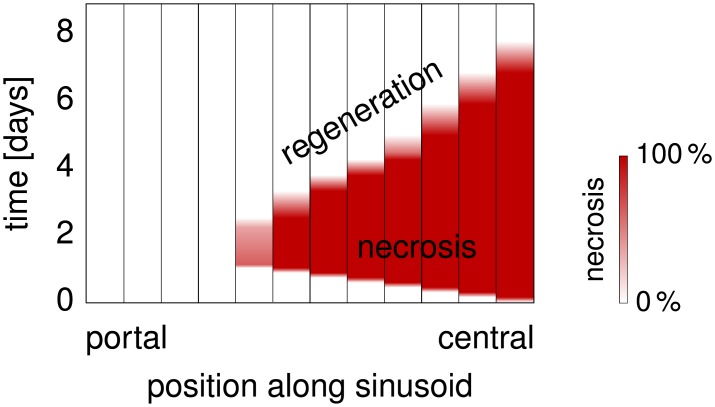
Necrosis and Regeneration. The plot shows how the spatial extent of the necrotic region evolves according to our model of the effect of CCl_4_ intoxication along a representative sinusoid of a mouse liver. The representative hepatocytes are separated by vertical black lines in this plot, A color range from white to red indicates zero to full necrotic damage of the respective representative hepatocyte. Necrosis develops during the first day, until a maximally necrotic state is attained. Subsequent regeneration starting on the second day leads to a shrinkage of the necrotic region until the end of day 7.

**Synthetic Necrosis Data:** A CCl_4_ dose of 1.6 mg g^−1^ body weight leads to necrotic areas reported in Fig 1S (manually derived data) in the supporting material to [34], with piecewise affine-linear interpolation in time and the assumption of full regeneration after 7 days. We use this data as the basis for defining a time-dependent necrotic zone in our representative sinusoids, converting, as explained in Eq 16, from pericentral area as visible in the histological images to pericentral length in the representative sinusoid model. The evolution of the necrotic area as input for our simulations is shown in Fig 6. A necrosis value between 0% and 100% is proportionally mapped to a change of volume fractions as described above.

## Applications and Results

We now show three example applications using our four-scale modeling and simulation framework. These applications were chosen to cover a wide range of species, compounds, pathological conditions, simulation scenarios, and pharmacologically and physiologically relevant output quantities. We first considered the clearance of midazolam in a healthy and a steatotic human liver where both zonation within sinusoids and organ-scale heterogeneity of the steatosis were present. Next, we addressed the clearance of caffeine in mice where the metabolization was zonated already in healthy livers. During the regeneration after CCl_4_ intoxication, which induces a zonal necrosis, an additional zonal pathophysiological change of the metabolization was taken into account, leading to different half-lives of the same amount of caffeine in the body. Finally, we show results of a parameter study investigating how sinusoid-scale spatially heterogeneous patterns of different cell entities influenced the uptake of insulin in mouse livers, representing the cell variation along a single sinusoid. Here, we evaluated how physiologically released periodic pulses were damped by the liver.

The midazolam and caffeine models are of the structure presented in Eq 3. The parameters needed therein and in Eq 4 for the two compounds are listed in Table 2. The insulin model, in contrast, has a different structure presented below in Eq 21. It was moreover parametrized using experimental ex vivo data for cells in a non-flowing medium, requiring a model and parameter conversion as described above for being able to use the model in our representative sinusoid approach. The steatotic perturbation in the midazolam model involved an organ-scale heterogeneity and was thus investigated using a realistic geometric model of a human liver and its vascular structures. The other two examples considered inhomogeneity only at the lobular scale and thus did not require organ-scale geometric models.

**Table 2:** Pharmacokinetics Parameters. For the two compounds whose pharmacokinetics is described by Eqs 3 and 4, the table lists the respective PK parameters: partition coefficients *κ*, permeabilities *P*, metabolization parameters $V_{\text{max}}^{\text{cell}}$ and $K_{m}^{\text{cell}}$; the lipophilicity log *P*

Compound Species	**Midazolam Human**	**Caffeine Mouse**
*κ*_rbc,pls_ [–]	3.903 ⋅ 10^−1^	5.850 ⋅ 10^−1^
*κ*_pls,int_ [–]	3.842 ⋅ 10^−1^	8.735 ⋅ 10^−1^
*κ*_int_ [–]	6.246 ⋅ 10^−2^	9.731 ⋅ 10^−1^
*κ*_cell_ [–]	1.060 ⋅ 10^−2^	1.192 ⋅ 10^0^
*P*_rbc,pls_ [s^−1^]	9.871 ⋅ 10^−3^	4.017 ⋅ 10^−3^
*P*_pls,int_ [s^−1^]	6.460 ⋅ 10^0^	1.548 ⋅ 10^2^
*P*_int,cell_ [s^−1^]	6.776 ⋅ 10^0^	1.039 ⋅ 10^0^
$V_{\text{max}}^{\text{cell}}$ [mM s^−1^]	2.531 ⋅ 10^−7^	6.609 ⋅ 10^−3^
$K_{m}^{\text{cell}}$ [mM]	1.0 ⋅ 10^−8^	4.52 ⋅ 10^−1^
log *P* [–]	3.107 ⋅ 10^0^	−7.0 ⋅ 10^−2^

### Midazolam Metabolism of Human Steatotic Livers

Midazolam is a sedative and anesthetic induction agent [116]. It is metabolized by the enzyme CYP3A4 [117] only expressed in the pericentral region [118].

#### Pharmacokinetics Model

Based on Fig 1 in [119], we estimated the size of the pericentral region of CYP3A4 expression to be be slightly over 50% of the lobular cross-section area. By Eq 16, this pericentral area corresponds to 14 of 19 hepatocytes performing the metabolization.

We used the available software [59] to establish a human PBPK model for midazolam based on physiochemical parameters from [120] and the Human Metabolome Database [121] as well as to determine the partition coefficients *κ* and the permeabilities *P* listed in Table 2. The parameters $V_{\text{max}}^{\text{cell}}$, $K_{m}^{\text{cell}}$, and log *P* were fitted to data manually derived from Fig 2 in [122].

This model was parametrized for a 19-zonal liver model matching our representative sinusoid model for humans, the parameters could thus be used without further adaption.

#### Simulation Results: Influence of the Body Delay

First, we represented the healthy liver by a single representative sinusoid and used an intravenous bolus injection of 11.42 mg as in the experiments underlying the data used above for fitting. Here, we determined the simulated midazolam concentrations in the plasma entering the liver via the portal vein, comparing a recirculation model as described above to one without delay during the recirculation.

**Fig 7:**
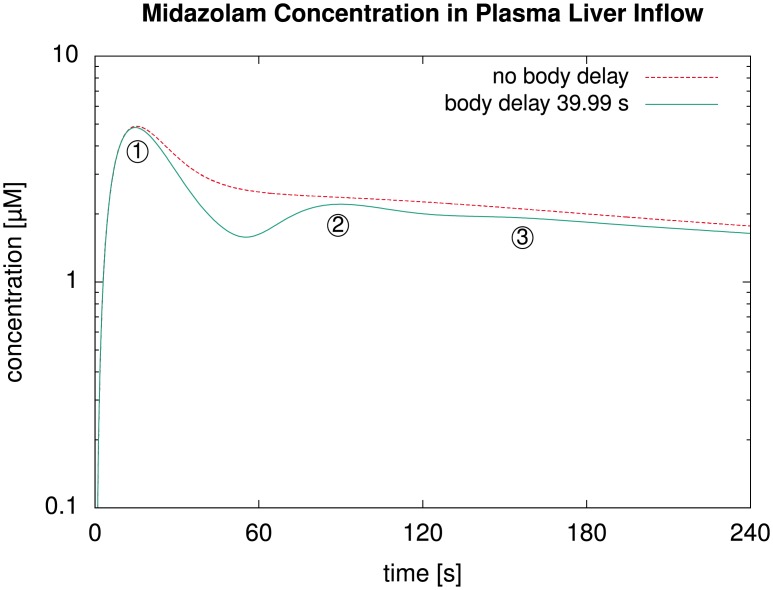
Influence of the Body Delay. For an intravenous bolus injection in our human whole-body model with a liver described by a single representative sinusoid, the plot shows the simulated midazolam concentration in the blood plasma at the liver inflow. Setting the temporal delay of the recirculation to zero (dashed line) shows that the delay in the model is indispensable for a correct prediction of recurring peaks for a second and third pass, indicated by circled numbers in the plot.

Results for this simulation in Fig 7 show that, without recirculation delay, our model failed to predict reoccurring peaks of the second and third pass. With nonzero recirculation delay, these peaks were captured with a physiologically reasonable temporal delay. In our case, we observed a temporal spacing of about 75 seconds between the first two peaks. This is clearly longer than a time span of approximately 23 s which can be estimated from Fig 4 in [63]. The literature data is for a different compound with potentially different PK characteristics throughout the body, which could account for part of the difference. Mainly, the difference is due to our recirculation model not yet accurately representing the flow delays by different organs and for different paths through the body. However, a more accurate recirculation delay is beyond the scope of this article where we focus on a detailed liver model and consider the simplified recirculation delay sufficient for our purposes. In particular, we did not simply pick our recirculation delay *τ*_rec total_ from Eq 1 such that the time span between two peaks matches the literature values. Such an approach would merely hide the inaccuracy described and would probably lead to incorrect amplitude of the second peak because flow delays by slower paths through the body would not be represented correctly and the circulation speed of the entire mass would be overestimated.

#### Results for the Zonated, Organ-Scale Homogeneous Model

We next assumed infusion of the same dose of midazolam into the blood plasma flowing through the portal vein within 5 seconds. In this case, we determined the simulated spatio-temporally resolved concentration profiles along the sinusoid in the healthy, predominantly periportal, predominantly pericentral, and non-zonal steatotic cases.

**Fig 8:**
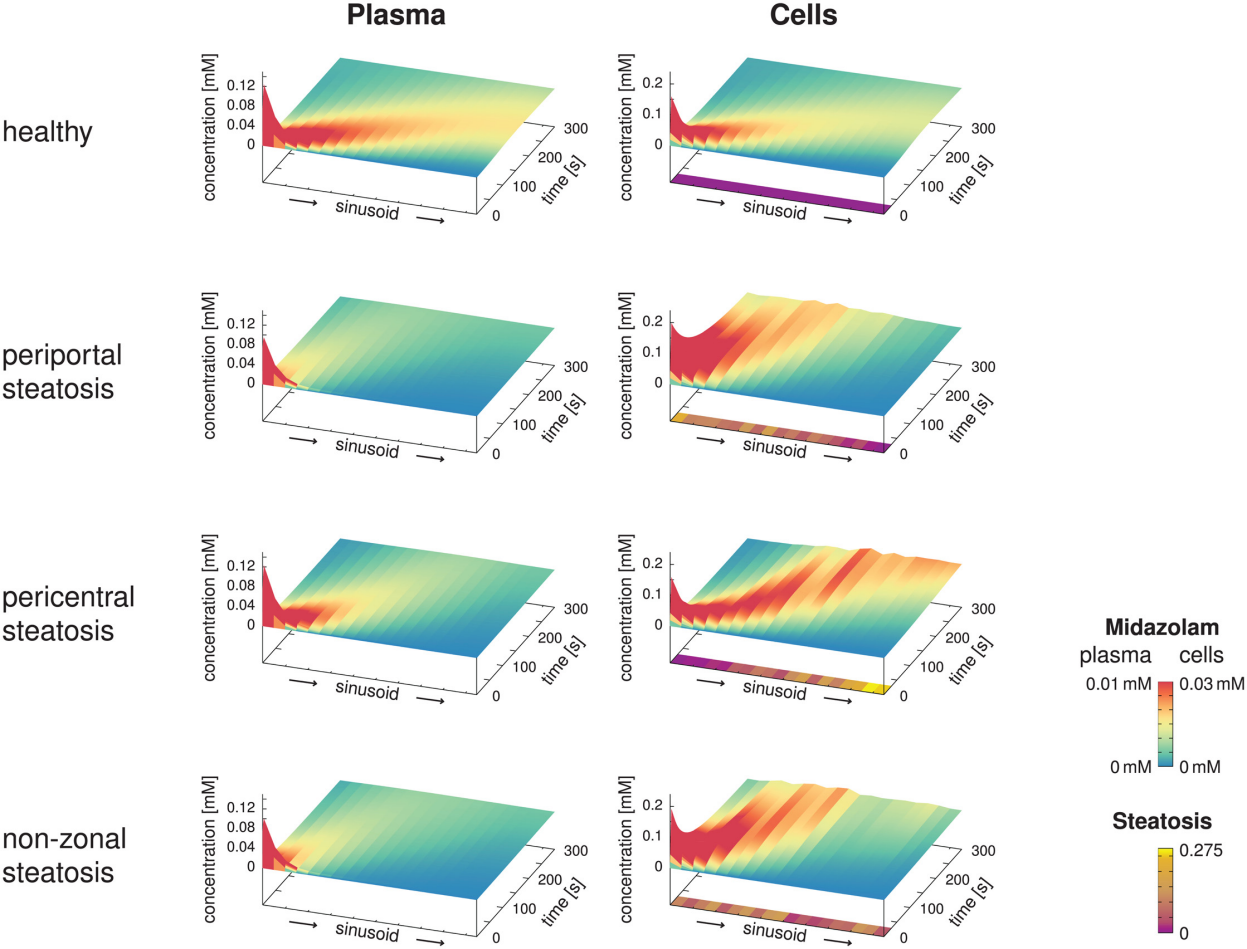
Spatio-Temporal Midazolam Concentration Profiles. The surface plots show the spatio-temporal evolution of the midazolam concentrations in the blood plasma and the hepatocytes along representative sinusoids assuming an infusion of duration 5 seconds into the portal vein, comparing the healthy reference case with three different steatotic cases with the same total amount of lipid accumulation. While the height in the graph covers the total concentration ranges, the color highlights differences in a lower range of concentrations, emphasizing the differences between the four cases. In addition, the steatosis patterns along the sinusoids are shown below the cellular concentrations. Differences in the transit time of the peak are due to different extent of storage and release of the midazolam due to the steatotic lipid accumulations. This should not be mistaken for the blood flow transit time, which is 13.6 s for all four cases and thus much shorter than the peak transit time.

In Fig 8, we can clearly observe the influence of the different steatosis patterns on the spatio-temporal midazolam distribution. As expected from Eq 17, a higher accumulation of midazolam was predicted for the steatotic regions. In the plots, the apparent velocity of the peak is particularly noteworthy with an apparent transit time of about 200 seconds for the healthy state, and slower apparent velocity for the steatotic states. We emphasize that this time scale is different from the blood flow velocity, for which the transit time is 13.6 s as given in Eq 6, i.e., significantly shorter and according to our assumptions in particular independent of the steatosis.

The simulation results here clearly depend on how the randomized steatosis patterns were chosen, i.e., on the seed value of the pseudo-random number generator. While it would be reasonable to investigate how sensitive the results are to the seed, and more generally to all the model parameters, such a sensitivity analysis is beyond the scope of the present exemplary use case.

#### Results for the Zonated, Organ-Scale Heterogeneous Model

Moreover, we considered a liver model using 10 000 representative sinusoids with the vasculature as shown in Fig 3. In this case, each representative sinusoid had its individual steatosis pattern (predominantly periportal predominantly pericentral, non-zonal), additionally the lateral organ-scale gradient as given in Eq 19 was present. Due to the large number of sinusoids, the influence of the individual patterns, as discussed above, averaged out. For these simulations, we again assumed an infusion into the blood plasma flowing through the portal vein within 5 seconds. We then determined the simulated midazolam concentrations in the systemic venous blood plasma. We again compared healthy and the three steatotic cases, where we now additionally considered an organ-scale heterogeneity of the steatosis according to Eq 19.

**Fig 9:**
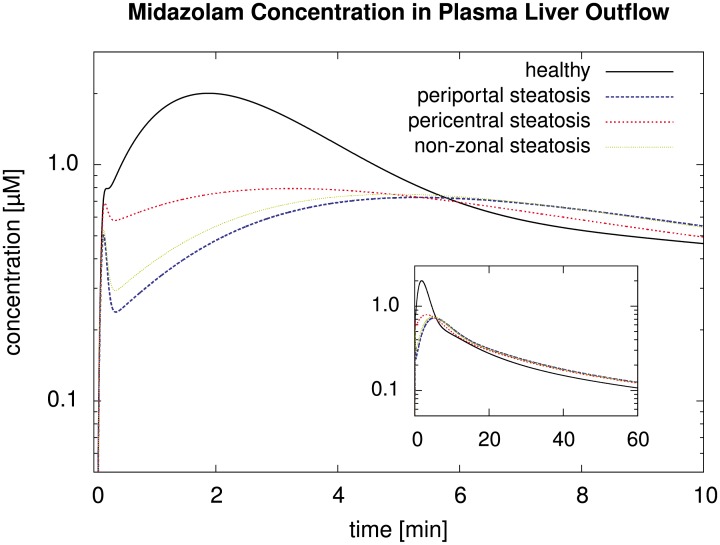
Organ-Scale Midazolam Concentration Profiles. For an assumed infusion of midazolam into the portal vein within 5 seconds, and our human whole-body model with a liver described by 10 000 representative sinusoids, the plot shows the midazolam concentrations in the blood plasma flowing out of the liver. In these model predictions, the different steatotic cases lead to differences during the first minutes. Afterwards, only the healthy state leads to concentrations distinct from the steatotic cases.

In Fig 9, differences between the three steatotic states during the first minutes can be observed. This shows a key property of our spatially resolved model which is capable of distinguishing different spatial patterns with the same total steatotic lipid accumulation. In these liver outflow curves, two concentration maxima can be observed. The first peak corresponds to the first pass after the blood flow transit time of the organ, which is the same for all four cases. The second peak corresponds to the different apparent peak velocities for the four cases observed in Fig 8. This shows that the effect of different apparent peak velocities is also present in the superposition of 10 000 different steatosis patterns of the four types (healthy, predominantly periportal, predominantly pericentral, non-zonal) and in particular not an artifact of the specific single patterns used for the simulations shown in Fig 8.

For longer simulations, only the difference between the healthy and steatotic cases can be observed. However, the quantitative difference mainly depends on the extent of steatosis, which is not the focus here. Still, the relatively small difference between the healthy and the steatotic states fits to the clinical finding in [123] that morbid obesity does not lead to changes in Midazolam clearance. Despite the qualitative similarity, this comparison is clearly limited. While obesity and steatosis are associated, the latter was not reported for the individuals considered in [123]. Moreover, our model did not take into account any aspects of obesity besides the hepatic steatosis.

### Caffeine Metabolism of Regenerating Mouse Livers

Caffeine is a central nervous system stimulant naturally occurring in some plants and it is one of the most widely consumed xenobiotics [124]. Pharmaceutically, it is commonly used in combination with analgesics [125] and antihistamines, but also for treatment of apnoea in premature infants [126].

#### Pharmacokinetics Model

For caffeine, we used the PBPK model from [19] determined for an intravenous bolus injection of 5 mg caffeine per kg body weight. Caffeine is metabolized by Cyp1a2 [127], an enzyme only expressed in the pericentral region [12], which we estimated to be about 58% of the lobular cross-section area based on Fig 3 in [128]. By Eq 16, this area of enzyme expression was translated to 9 of 12 pericentral hepatocytes performing the metabolization, the 3 periportal hepatocytes do not. We hence used the enzyme activity parameters from the PBPK model assigned above for the 9 pericentral hepatocytes and set the term *m* from Eq 2 to zero for the 3 periportal hepatocytes. The intra-subcompartmental exchange took place in all 12 representative hepatocytes in the model, i.e., *E*(*λ*) from Eq 15 is present for all of them. Despite a direct zonated translation to the hepatocytes, an adaption from well-stirred to representative sinusoid models as described above turned out not to be necessary for this model.

Regeneration after CCl_4_ intoxication was built into the model as described above by synthetic necrosis data. The necrotic region was also located pericentrally and at its maximum size covered most of the metabolizing region. Besides those two zonated effects, no additional organ-scale heterogeneity was present in the model, it is thus sufficient to represent the liver by one representative sinusoid. This model included recirculation with a recirculation delay of *τ*_body_ = 15.5 seconds as explained above.

#### Simulation Results

In our simulations, we considered an intravenous bolus injection of 5 mg caffeine per kg body weight, as in the underlying experimental data of the model. The injection was assumed to take place at different time points after inducing CCl_4_ damage, from 0 days, when necrosis starts to develop, to 7 days, when the fully recovered state according to our damage model is attained. After the injection, we simulated one day of blood flow and caffeine clearance in the model, involving spatially and temporally resolved caffeine concentrations for the representative sinusoid and temporally resolved concentrations for the other compartments in our body-scale model. From these values, we computed the total amount of caffeine present in the body and in particular the half-life *t*_1/2_, i.e., the time it took for half of the initial caffeine to be cleared from the body, cf. elimination half-life in [129]. While this half-life is easy to compute in the model by summing up caffeine amounts in all compartments, it is not easily accessible experimentally. Instead, many studies determine the half-life of the compound in the systemic blood plasma, leading to slightly different results.

**Fig 10:**
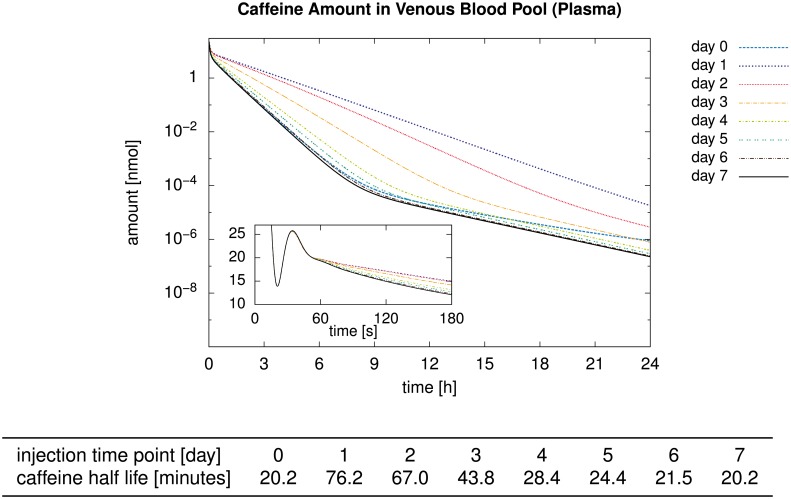
Caffeine Clearance After CCl_4_-Induced Necrosis. The plot shows the caffeine concentrations in the plasma in the venous blood pool for 24 hours after intravenous bolus injection at different time points during the necrosis development and regeneration process induced by CCl4. The influence of the necrotic damage occurred during day 0, leading to slowest decrease of caffeine concentration in the venous blood for the injection at day 1. Regeneration during days 1 to 7, according to our necrosis model from Fig 6, lead to increasingly faster decrease of the caffeine concentrations for the later injections, until the fully regenerated state was reached for the injection at day 7. The inset shows the second pass after the first recirculation cycle, in this case after 30.2 s. The table lists the half-life of caffeine in the organism, i.e., the first decrease by 50%, if it occurs within one day after the administration. These numbers confirm the observations from the plot which only involves the venous blood pool.

The caffeine concentrations in the plasma in the venous blood pool show a second and weak third peak after about 30 and 60 seconds, see the small line plot in Fig 10. Later peaks are of diminishing amplitude and not visible in the curves. These recurring peaks are superpositions of the delays due to the liver model and the recirculation as well as an exchange between subcompartments of all organs, the spatially resolved liver and the well-stirred remaining organs. The peaks thus take longer than the total recirculation time for mice of *τ*_rec total_ = 19.72 s, see the discussion of the recirculation time scale in the human midazolam case above. This effect qualitatively occured regardless of the necrotic state of the model. On the longer time scale of one day, the necrotic state of the model had a major influence, see large line plot in Fig 10: for injection at day 0, the necrosis developed and slowed down the decrease of caffeine concentration in the plasma in the venous blood pool. For injection at day 1, the slowest concentration decrease can be observed. Regeneration lead to an increasingly larger metabolizing region for the injections at days 2 to 7, leading to faster decrease of the caffeine concentrations, until the fully regenerated state was reached for the injection at day 7.

Correspondingly, a change in the half-lives listed in Fig 10 can be observed. The maximally necrotic state at day 1 (*t*_1/2_ = 76.2 minutes) and the healthy state at day 7 (*t*_1/2_ = 20.2 minutes) differ by a factor of 3.77. This fits to the experimental findings in [130]. These experiments involved rats one day after inducing necrosis by CCl_4_ compared to control rats, thus a different species. Furthermore, different doses of 0.25 mg CCl_4_ per kg body weight and 5 mg caffeine per kg body weight were used as well as a different notion of half-life computed based on concentrations in the systemic blood plasma. Still, the half-lives reported in Table 1 in [130] are 1.68 hours (control) and 6.29 hours (day 1) and thus differ by a factor of 3.74, surprisingly close to our finding. Computing half-lives in a similar way as in [130], i.e., via an elimination rate constant obtained by logarithmic regression analysis to the concentration in the venous blood pool in the range [30, 300] minutes, we obtain 28.5 and 76.8 minutes, respectively. This is, on the one hand, in the same range as our half-lives *t*_1/2_ for the entire body. On the other hand, they differ by a factor of 2.69, which is again close to 3.74 obtained from [130].

### Murine Insulin Uptake: A Parameter Study

The hormone insulin plays an important role for the regulation of the glucose and lipid metabolism [131]. In healthy organisms, insulin is secreted by the *β* cells of the pancreas into the portal vein in periodic, discrete pulses between 5 and 15 minutes [132]. The liver is the main organ involved in the insulin degradation and its depletion from the blood. Of the secreted insulin, 40% to 80% are extracted hepatically before reaching other organs [133]. This extraction can be observed in terms of the amplitude as well as the integral of inflowing and outflowing insulin concentrations in the hepatic blood flow, see, e.g., the data reported in Fig 2 in [134] for rats and in Fig 1 in [135] for dogs. The high insulin clearance by the liver during the first pass leads to a prominent periportal to pericentral gradient of the insulin concentration in the sinusoid. The effect of the periodicity of the pulses in the hepatocytes is not well known, but non-pulsatile insulin secretion is a well-known early indicator of type 2 diabetes, indicating a predominant role in the maintenance of biological functions [136, 137].

**Fig 11:**
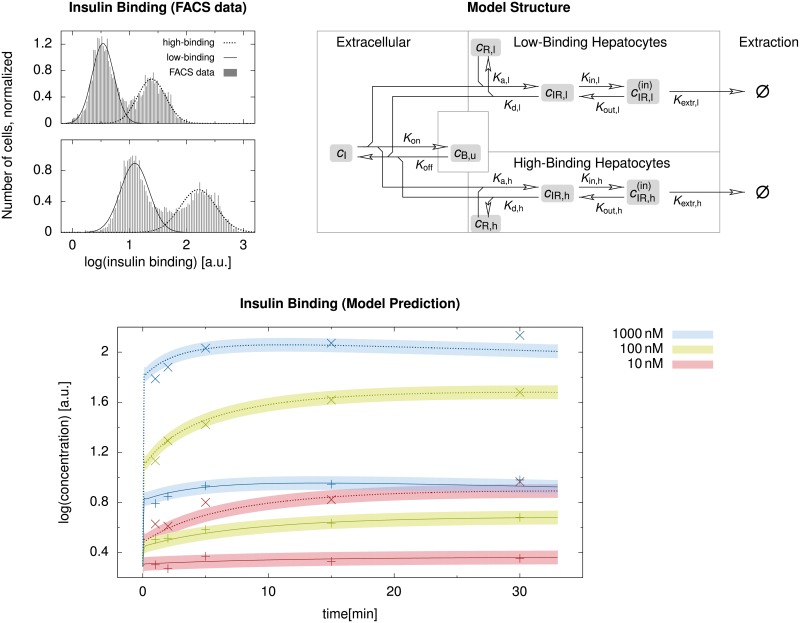
Insulin Model. The *top left* plots show two datasets for evaluating the amount of insulin binding to individual cells by flow cytometry (FACS). The upper panel exemplarily shows the raw measurements five minutes after stimulation with 100 nM, the lower panel for 1000 nM. Independent of time and insulin dose, a bimodal distribution was observed indicating two entities of hepatocytes. Two Gaussian distributions were fitted to the logarithmic intensity histograms to analyze the time and dose dependency of the average insulin binding within both entities. The *top right* sketch shows the structure of the model for cellular insulin binding, internalization and extraction processes described by Eq 21 including the parameters given in Eq 22. This model could explain the dynamics for both entities of hepatocytes as seen in the *bottom* plot showing the model fit of time-dependent average insulin binding of both entities for three different doses. The experimentally observed data for low-binding and high-binding hepatocytes is shown as ‘+’ and ‘×’, respectively. Model predictions for these cases are solid and dashed lines, respectively. The shaded error bands correspond to the estimated error in the data points.

Due to the hepatic insulin degradation, the hepatocytes face much larger insulin amounts than other cells in the body. We experimentally found two entities of hepatocytes differing in their abilities to bind and endocyte insulin, see Chapter 3 in [138] and Fig 11. These two entities of hepatocytes are denoted as *low-binding* and *high-binding* in the following. Our first experimental observations do not indicate that there is a noticeable zonation of the two cell entities along the sinusoids.

We here used the simulation techniques introduced above to predict the impact of different spatial patterns of low-binding and high-binding cells on the pulsatility of insulin in the blood flowing out of the liver. Using experimental data obtained ex vivo with freshly isolated hepatocytes, we developed a mathematical model for binding and endocytosis of insulin for the two cell entities at the cellular level. This model is integrated in the representative sinusoids to be able to study the effect of different spatial patterns formed by the two entities of cells. The fractions of the number of cells are denoted by *η*_l_ and *η*_h_ = 1 − *η*_l_ for the low- and high-binding entities, respectively. We assume that both cell entities have the same size, so that *η*_l_ and *η*_h_ can also be interpreted as the respective volume fractions. In our experiments, we observed a fraction *η*_l,obs_ = 0.606 of low-binding cells. As literature data for measurements of insulin concentrations flowing into and out of the liver are used for comparison, we here consider an isolated perfused liver [139], omitting recirculation and thus the body-scale in our model.

The model for cellular binding of insulin correctly describes the dependency on dose and time observed experimentally for both entities of hepatocytes without considering the intracellular pathway. This indicates that insulin binding and degradation is not strongly regulated by intracellular pathway feedbacks. Thus, we did not take into account intracellular signalling processes involved in insulin uptake such as those in [140]. Similarly, the experiment did not involve extracellular processes such as those considered in [141], these were also omitted in the model. We are aware that such an in vitro to in vivo extrapolation is a highly non-trivial task and involves many sources of uncertainty, see [142, 143] and particularly [144] for hepatocytes, but consider the model appropriate for the proof of concept application here. In particular, detailed biological interpretation, discussion of parameter uncertainty and parameter sensitivity is beyond the scope of this example application.

#### Insulin Model

The dynamic model of cellular insulin binding consists of eight ordinary differential equations describing the processes shown in Fig 11 by chemical mass action laws. For parameter estimation, flow cytometry (FACS) measurements of labelled insulin were used. The dose-response 15 minutes after stimulation as well as time course data (*t* = 0, 1, 2, 5, 15, 30 min) for 10 nM, 100 nM and 1000 nM were repeatedly measured and averaged [138]. A selection of the FACS data is shown Fig 11. In addition, the dose- and time dependency of insulin depletion in the medium was evaluated by an enzyme-linked immunosorbent assay (ELISA).

The structure of the insulin model is different from the previous two models. The extracellular medium from the experimental setup is replaced in the simulations by the plasma subject to blood flow. The plasma is assumed to be in instantaneous concentration equilibrium with the interstitium (not subject to blood flow). Moreover, red blood cells are here assumed not to be part of the insulin exchange and we thus have no insulin concentration for the red blood cells in our model. This allows us to define an extracellular volume fraction *φ*_ext_ = *φ*_pls_ + *φ*_int_ where *φ*_pls_ and *φ*_int_ are given in Eq 5b. According to these flow assumptions, we scaled the typical transit time as *τ*_typ_ ⋅ *φ*_ext_/*φ*_pls_ = 15.3 s.

To account for the two different entities of hepatocytes, the cellular subcompartment consists of two volumes *φ*_cell,l_ = *η*_l_ *φ*_cell_ and *φ*_cell,h_ = *η*_h_ *φ*_cell_ for the low-binding and high-binding cells, respectively, that add up to *φ*_cell_ from Eq 5b. Additional processes such as insulin binding by receptors require further concentrations in the model, leading to a form of the equations different from Eqs 3 and 4.

Parameters in the cellular insulin model were originally fitted for the experimental setting with an unphysiological ratio of extracellular to cellular volume. This fit is shown in Fig 11. The parameters were subsequently rescaled and converted to the volume fractions in the physiological setting. For scaling the parameters derived in the well-stirred experimental environment to spatially resolved representative sinusoids, we applied the procedure described above. For this purpose, we first implemented a preliminary organ-scale model by introducing a flow term as the *α* in Eq 2 and defined a preliminary representative sinusoid model using these parameters. For this purpose, the position-dependent volume fractions *ψ*_ext_, *ψ*_cell,l_, and *ψ*_cell,h_ are computed in terms of the *ψ*_{pls,int,cell}_ defined in Eq 12 as

$$\begin{array}{cc} \psi_{\text{ext}}\left(\lambda \right) & =\psi_{\text{pls}}\left(\lambda \right)+\psi_{\text{int}}\left(\lambda \right)\\ \psi_{\text{cell,l}}\left(\lambda \right) & =\eta_{\text{l}}\psi_{\text{cell}}\left(\lambda \right)\\ \psi_{\text{cell,h}}\left(\lambda \right) & =\eta_{\text{h}}\psi_{\text{cell}}\left(\lambda \right)\;. \end{array}$$ (20)

We could observe that the receptor binding and unspecific binding are the fastest processes described by the ODE system. Hence, we determined an adaption factor *ω* for these binding parameters, considering the peak-above-baseline amplitude as the deviation to be minimized when determining an optimal *ω* = 0.204. This factor is part of the parameters *K*_{on,off}_ and *K*_{a,d},{l,h}_ in the final organ-scale model

∂tcI=(σ(λ)Koffψext(λ)cB,u-σ(λ)Konψext(λ)cI)+(σ(λ)·Kd,lψext(λ)cIR,l=(-σ(λ)·Ka,lψext(λ)cIcR,l)+(σ(λ)·Kd,hψext(λ)cIR,h-σ(λ)·Ka,hψext(λ)cIcR,h)∂tcB,u=σ(λ)Konψext(λ)cI-σ(λ)Koffψext(λ)cB,u∂tcR,l=σ(λ)·Kd,lψcell,l(λ)cIR,l-σ(λ)·Ka,lψcell,l(λ)cIcR,l+Kextr,lψcell,l(λ)cIR,l(in)∂tcR,h=σ(λ)·Kd,hψcell,h(λ)cIR,h-σ(λ)·Ka,hψcell,h(λ)cIcR,h+Kextr,hψcell,h(λ)cIR,h(in)∂tcIR,l=σ(λ)·Ka,lψcell,l(λ)cIcR,l-(σ(λ)·Kd,lψcell,l(λ)+Kin,lψcell,l(λ))cIR,l+Kout,lψcell,l(λ)cIR,l(in)∂tcIR,h=σ(λ)·Ka,hψcell,h(λ)cIcR,h-(σ(λ)·Kd,hψcell,h(λ)+Kin,hψcell,h(λ))cIR,h+Kout,hψcell,h(λ)cIR,h(in)∂tcIR,l(in)=Kin,lψcell,l(λ)cIR,l-(Kout,lψcell,l(λ)+Kextr,lψcell,l(λ))cIR,l(in)∂tcIR,h(in)=Kin,hψcell,h(λ)cIR,h-(Kout,hψcell,h(λ)+Kextr,hψcell,h(λ))cIR,h(in) (21)

where *c*_I_ denotes the concentration of free insulin in the plasma and interstitium, *c*_B,u_ the concentration of unspecifically bound insulin, *c*_R,{l,h}_ are the concentrations of insulin receptors, *c*_IR,{l,h}_ those of bound insulin and *c*_IR,{l,h}_ those of bound internalized insulin, the latter three in the low-binding and high-binding case, respectively. Permeability-type parameters are scaled as before using *σ*(*λ*) defined in Eq 14. The dependency of the concentrations on space and time was omitted here such as not to overload the notation. The relations between the concentrations are illustrated in the diagram in Fig 11.

The constants used in Eq 21 are

$$\begin{array}{cccc} K_{\text{off}} & =3.419\cdot 10^{-2}\;{\text{s}}^{-1}\\ K_{\text{on}} & =6.894\cdot 10^{-2}\;{\text{s}}^{-1}\\ K_{\text{d,l}} & =3.207\cdot 10^{-5}\;{\text{s}}^{-1} & K_{\text{d,h}} & =1.449\cdot 10^{-4}\;{\text{s}}^{-1}\\ K_{\text{a,l}} & =1.330\cdot 10^{-2}\;{\text{mM}}^{-1}\;{\text{s}}^{-1} & K_{\text{a,h}} & =4.242\cdot 10^{-2}\;{\text{mM}}^{-1}\;{\text{s}}^{-1}\\ K_{\text{in,l}} & =6.425\cdot 10^{-6}\;{\text{s}}^{-1} & K_{\text{in,h}} & =6.020\cdot 10^{-6}\;{\text{s}}^{-1}\\ K_{\text{out,l}} & =7.826\cdot 10^{-7}\;{\text{s}}^{-1} & K_{\text{out,h}} & =3.961\cdot 10^{-7}\;{\text{s}}^{-1}\\ K_{\text{extr,l}} & =1.893\cdot 10^{-7}\;{\text{s}}^{-1} & K_{\text{extr,h}} & =1.231\cdot 10^{-7}\;{\text{s}}^{-1} \end{array}$$ (22)

The unspecific binding expressed in Eq 21 by the concentration *c*_B,u_ is viewed as *(a)* free insulin which is *(b)* binding to the cells. Due to *(a)*, it is a concentration in the extracellular space, hence *ψ*_ext_(*λ*) in the denominator. Due to *(b)*, binding capability scales with the cellular surface area, hence the factor *σ*(*λ*) in the numerator.

#### Results of the Parameter Study

As a parameter study, we considered all 4096 possible configurations of 12 low- or high-binding cells along the representative sinusoid to investigate how the configuration affects the outflowing insulin concentration. As each position along the representative sinusoid represents a different contribution to the total volume, the factors *ρ*(*λ*) as defined in Eq 13 needed to be taken into account when computing the total fraction of the two cell entities for all possible configurations as *η*_{l,h}_ = ∑_*λ*_ *ρ*(*λ*) ⋅ *ψ*_cell,{l,h}_(*λ*). This in particular implies that there are more distinct values for *η*_{l,h}_ than merely multiples of 1/12. Many of the 4096 possible configurations lead to fractions of low-binding cells highly different from the experimentally observed ratio *η*_l,obs_ = 0.606. Even though we put special focus to ratios close to *η*_l,obs_ in the presentation of the results all possible combinations were studied to be able to comprehensively investigate the impact of spatial configurations.

Insulin secretion by the pancreas is pulsatile, where frequency and amplitude can vary and in particular depend on the organism and on the glucose level in the blood. The typical periodicity is in the range of 5 to 15 minutes [132]. We considered an inflowing plasma concentration composed of a basal level of 1 nM in both HA and PV and an oscillatory secretion component into the PV inflow with a period of 720 s (12 minutes) and a peak-above-baseline amplitude of 9 nM. This results in a total inflow of

$$I(t)=1\;\text{nM}+\frac{1.752}{2.102}\cdot \frac{9\;\text{nM}}{I_{\text{max}}}\cdot \left[(1-e^{-\text{ln}(2)/\tau_{1}\cdot t})e^{-\text{ln}(2)/\tau_{2}\cdot t}\right]\;t\in [0\text{s},720\text{s})$$ (23)

with half life times *τ*_1_ = 60 s and *τ*_2_ = 10.8 s and a scaling factor 1.752/2.102 being the relative contribution of the PV for the total blood flow [59]. The normalization factor *I*_max_ is given by the maximum

$$I_{\text{max}}=(1-\frac{\tau_{1}}{\tau_{1}+\tau_{2}})e^{\frac{\tau_{1}}{\tau_{2}}\ln(\frac{\tau_{1}}{\tau_{1}+\tau_{2}})}$$ (24)

of the unnormalized product of the two exponentials in square brackets in Eq 23. The profile *I*(*t*) was continued periodically for *t* ≥ 720 s. Its range of values is chosen to match the experimental setting for which the model was parametrized, it is in particular not meant to approximate physiological insulin concentrations.

Initial concentrations were obtained by running the simulation for the well-stirred organ-scale model for 1.8 ⋅ 10^7^ s (5000 hours). For this purpose, initial receptor concentrations of *c*_R,l_ = 5.686 ⋅ 10^−2^ mM and *c*_R,h_ = 1.366 ⋅ 10^0^ mM from the parameter fit and zero initial concentrations for the remaining states, as well as the average of *I*(*t*) from Eq 23 as inflow concentration were used. This resulted in

$$\begin{array}{cccc} c_{\text{I}}\left(0\right) & =1.353\cdot 10^{-6}\;\text{mM}\\ c_{\text{B,u}}\left(0\right) & =2.728\cdot 10^{-6}\;\text{mM}\\ c_{\text{R,l}}\left(0\right) & =5.662\cdot 10^{-2}\;\text{mM} & c_{\text{R,h}}\left(0\right) & =1.359\cdot 10^{0}\;\text{mM}\\ c_{\text{IR,l}}\left(0\right) & =3.150\cdot 10^{-5}\;\text{mM} & c_{\text{IR,h}}\left(0\right) & =5.370\cdot 10^{-4}\;\text{mM}\\ c_{\text{IRin,l}}\left(0\right) & =2.082\cdot 10^{-4}\;\text{mM} & c_{\text{IRin,h}}\left(0\right) & =6.227\cdot 10^{-3}\;\text{mM} \end{array}$$ (25)

**Fig 12:**
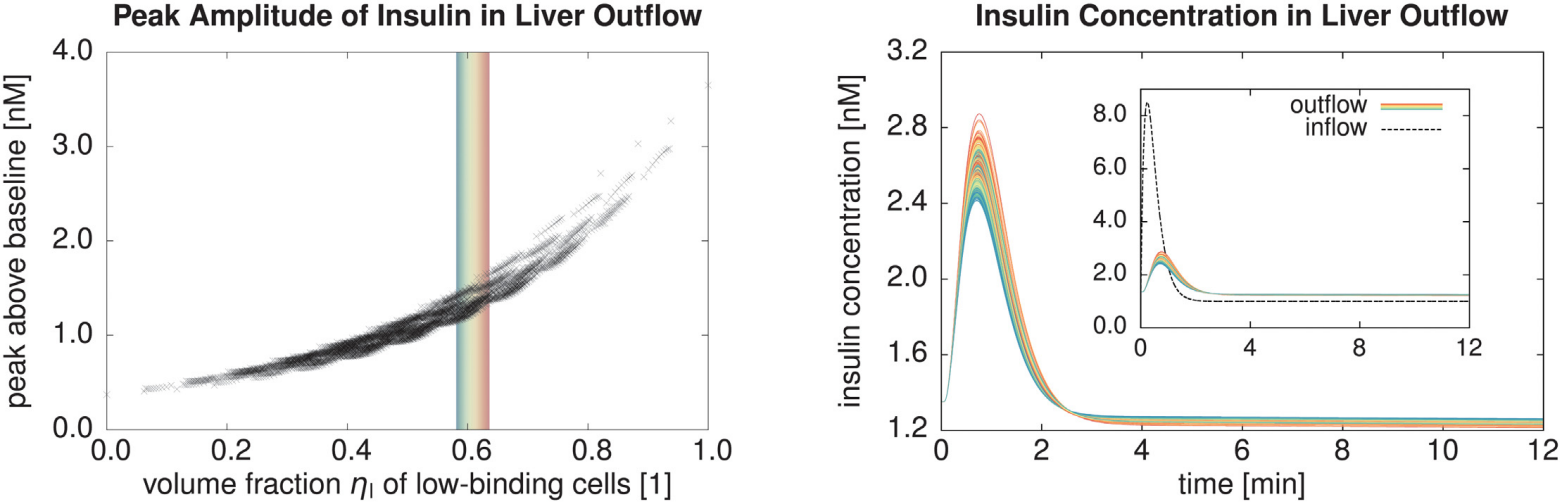
Parameter Study for the Insulin Model. The *left plot* shows the relation between the fraction of low-binding cellular volume and the peak-above-baseline amplitude of the outflowing insulin concentration obtained by the simulation for 4096 different spatial configurations of low-binding and high-binding cells. Generally, a higher fraction of low-binding cells implies less insulin uptake by the cells and thus a higher simulated outflowing concentration. The scattering, however, clearly shows that it is not a strict functional dependency. The shaded area in the scatter plot corresponds to the range *η*_l_ = 0.606 ± 0.025 near the observed fraction of low-binding cells. It comprises 436 cases, for which the corresponding curves of outflowing insulin concentration are shown in the *right plot*. The individual lines are colored according to the low-binding cellular volume, cf. the shaded area in the scatter plot. The fact that these are not spectrally ordered from blue to red again shows that the simulation result does not only depend on the low-binding cellular volume fraction but also on the actual spatial configuration.

In Fig 12, we show the dependency of the peak-above-baseline amplitude of the outflowing insulin concentration on the fraction of low-binding cells for all 4096 cases, i.e., for the full range of 0 ≤ *η*_l_ ≤ 1. Additionally, we plotted the outflowing concentration time curves for configurations with 0.581 ≤ *η*_l_ ≤ 0.631 low-binding cells, the range *η*_l,obs_ ± 0.025. We can observe that there is a relatively wide range of outflowing peak-above-baseline amplitudes. The obtained results indicate that not only the amount of the two entities of cells, but also their spatial configuration along the sinusoids determine the total insulin binding and degradation in the liver. Despite more experimental work being necessary in order to corroborate theses observations, it is clear that the insulin outflow from the liver is determined by the existence of both entities of cells able to bind different amounts of the hormone as well as by their spatial configuration along the sinusoid. The latter seems to be a new additional diversity factor in the liver independent from the well-known metabolic heterogeneity related to the position of the cells along the sinusoid.

## Discussion

In the three example applications, we considered two rather distinct cases of organ-scale heterogeneity. In the caffeine and insulin examples, only sinusoid-scale heterogeneity is present and so that the model consists of a single representative sinusoid. In contrast, our midazolam example is based on highly heterogeneous synthetic data at the organ scale and uses 10 000 representative sinusoids. For actual biological or pharmacological applications, the appropriate number of representative sinusoids and thus the model complexity should be chosen based on the available data on organ-scale heterogeneity of perfusion and parameters as well as the specific simulation output quantity of interest.

### Computational Performance

The computational performance for our example applications are summarized in Table 3 for single-threaded simulations on an Intel Core i7 2.8 GHz CPU, compiled using GCC 4.8.4. The performance clearly depends on the presence of the submodels, the lack of a recirculation ODE system in the insulin application clearly saves time and memory, but may also vary with the numerical properties of the equations. The midazolam example with 10 000 representative sinusoids has rather high organ-scale resolution, but can still be simulated in reasonable time on a standard desktop PC. In a previous, porous-medium-based approach, a simulation with merely 800 leaf nodes of the vascular systems was reported to take about 9 times as long (cf. Table 1 in the supporting information Text S1 of [8]), albeit for a different compound in a mouse liver and on slightly slower hardware. For high organ-scale resolutions, the memory requirement and computational performance essentially scale linearly with the number of representative sinusoids considered.

**Table 3:** Computational Performance. For our applications with different numbers # rs of representative sinusoids, the table lists the computational performance as a multiple of real-time performance (larger is faster) as well as the memory needed for the respective simulation. For the insulin model, the number refers to one of the 4096 simulations run as part of the parameter study.

application	# rs	simulated / CPU Time [1]	memory [MiB]
midazolam	1	96.919	45
midazolam	10000	0.033	2518
caffeine	1	26.643	68
insulin	1	2441.017	48

### Limitations

Besides limitations already addressed at the appropriate sections above, a number of general limitations of the approach presented in this article should be mentioned. First and foremost, obtaining targeted experimental data to validate our simulation results is beyond the scope of the present article. The comparison to literature data for the midazolam and caffeine examples above are only a first, albeit successful, step in this direction. In the course of performing appropriate wet lab and numerical experiments for validation, we may need to refine certain assumptions as part of our model simplifications and reduction of dimensions, such as that from the 3D lobuli to the 1D representative sinusoids. Due to the modular structure of the model and its implementation, we expect this to pose no major challenge. Moreover, our refinement of whole-body PBPK models by adding spatial resolution has clearly focused on one organ, the liver. We thus neglect the potential influence of other organs on the clearance of other compounds, see the examples listed in [145]. While certain concepts presented here may be transferable, details about modeling the processes taking place in other organs will require a thorough investigation. As for the steatosis and necrosis models used above, these already contain strong simplifications for the liver. More realistic models should take into account the relevant influences, for all organs, on the pharmacokinetics of the compound being considered. Besides modeling, a major challenge is to experimentally obtain data for appropriately quantifying these influences. While the model perturbations above were based on synthetic data, it is possible to use experimentally observed patterns if such data is available, e.g., quantifying the sinusoid-scale and organ-scale heterogeneity of steatosis patterns as shown in Fig 1, see preliminary results in [146] and [147]. Similar steatosis patterns could then be clustered appropriately to choose the number of representative sinusoids, i.e., the required complexity of the model for sufficient accuracy of the simulation results.

### Outlook

Experimental validation could involve systematic pharmacokinetic measurements with an isolated perfused liver including partial bypassing and retrograde and antegrade drug perfusion as, e.g., performed in [34]. Besides a thorough validation, an uncertainty and parameter sensitivity analysis is needed to identify critical and less critical parameters, see [148] for an overview of sources of variability in PK models. For this purpose, being able to run fast simulations is an important starting point. An application of the modeling framework described here for other compounds, species, pathologies, etc., mainly requires the appropriate model parameters determined for the specific application. Further model integration [149] should involve including cellular metabolic models such as described in [37] and investigating how numerically homogenized model parameters can be employed from or provided to alternative approaches like [8, 34, 37, 52].

### Conclusion

We have presented a generic multiscale physiology-based simulation framework for perfusion and metabolization in the liver which can deal with inhomogeneity at distinct spatial scales. It permits high temporal resolution to investigate first-pass effects as well efficiency for simulating long periods of time and performing parameter studies. Three applications show that this is a versatile approach applicable to diverse pharmacological questions, in particular investigating the impact of zonation and organ-scale heterogeneous pathophysiological changes.

## Supporting Information

S1 Dataset Geometric Liver Model. The geometry information used for the human midazolam simulations, i.e., the organ mask and vascular tree datasets shown in Fig 3, are provided as an online resource. A simple viewer tool for the vascular trees from the supporting information Data S1 of [8] is included.
(ZIP)
